# Photosynthesis-informed breeding to enhance Soybean (*Glycine max*) yield potential: a framework integrating high-throughput phenomics, genomic prediction and G×E×M

**DOI:** 10.1007/s00122-026-05374-8

**Published:** 2026-09-29

**Authors:** Harmeet Singh-Bakala, Francia Ravelombola, Caio Canella Vieira, Laavanya Rayaprolu, Matthew Herritt, Jacob D. Washburn, Ru Zhang, Feng Lin, Grover Shannon

**Affiliations:** 1 University of Missouri-T.E. Jake Fisher Delta Research, Extension and Education Center, Portageville, MO 63873 USA; 2 Division of Plant Science and Technology, University of Missouri-Columbia, Columbia, MO 65202 USA; 3 Division of Agriculture, Department of Crop, Soil, and Environmental Sciences, University of Arkansas, Fayetteville, AR 72701 USA; 4 USDA-ARS, U.S. Arid Land Agricultural Research Center, Maricopa, AZ 85138 USA; 5 Plant Genetics Research Unit, USDA-ARS, Columbia, MO 65201 USA; 6 Donald Danforth Plant Science Center, St. Louis, MO 63132 USA

## Abstract

**Key message:**

Unexploited genetic variation in soybean photosynthetic efficiency can be converted into yield gains by coupling high-throughput phenotyping with genomic-phenomic prediction within a G×E×M-oriented breeding framework built on heritable, physiologically meaningful traits.

**Abstract:**

Improving crop yield through enhanced photosynthetic efficiency (PE) is a re-emerging and increasingly tractable objective for modern breeding, driven by recent advances in high-throughput field phenotyping, genomics, and data analytics. In C3 crops like soybean, radiation use efficiency (RUE) remains below the theoretical maximum, and evidence from cereals and legumes indicates that gains in RUE have contributed substantially to long-term yield improvement, suggesting unrealized potential in soybean. However, despite extensive physiological and genetic studies, PE has historically been difficult to integrate into breeding programs due to the complexity of photosynthetic processes and the historical lack of reliable high-throughput field-scale phenotyping and predictive breeding frameworks. This review synthesizes current knowledge on biological determinants of PE in soybean (leaf and canopy morphology, non-foliar photosynthesis), alongside natural genetic variation and the genetic architecture of photosynthesis (PS)-related traits. We summarize recent transgenic and functional studies that demonstrate the capacity to modify PS processes, while highlighting challenges of achieving durable yield benefits in real field conditions. We further review emerging high-throughput phenotyping (HTP) platforms, including unmanned aerial vehicles (UAVs)-assisted multi/hyperspectral imaging and ground-based portable sensors, that enable scalable assessment of PS-related traits across diverse field environments and management regimes. We argue that bridging the gap between photosynthetic potential and realized yield requires the integrating HTP with genomics, phenomic prediction and molecular breeding frameworks. We propose a pragmatic breeding pipeline that prioritizes standardized, biologically meaningful and heritable PE traits and proxy traits, leverages diverse germplasm for allele discovery, and applies integrated genomic-phenomic prediction frameworks that explicitly incorporate genotype × environment × management (G×E×M) interactions. By embedding PS-traits as complementary selection criteria within breeding pipelines, this multi-disciplinary approach offers a practical route to accelerate genetic gain and enhance soybean yield resilience.

## Introduction

Soybean [*Glycine max* (L.) Merr.] is the leading oilseed crop around the globe. Economically, it is the most important oilseed as it accounts for more than 60% of vegetable oil production and is the largest plant-based protein source in the world (Graham and Vance 2003; Martignone et al. 2024; Rotundo et al. 2024). Its significantly higher seed protein content makes it more nutritious for consumption compared to cereals like wheat, rice, and maize. During 2024–25, soybean was cultivated at 146.8 million hectares (mh) with a global production of 424.2 million metric tons (mt), of which 118.8 million mt were in the US across 34.8 mh. The yield productivity stood at 2.9 metric tons per hectare (3.4 mt/ha in the US) (USDA 2025). About 80% of world soybean production comes from Brazil, USA, and Argentina (USDA 2025). Despite decades of significant genetic gains, the rate of yield improvement in soybean has plateaued relative to other major crops, largely due to narrow genetic base (Hyten et al. 2006; Mikel et al. 2010; Kofsky et al. 2018; Bayer et al. 2022). Also, there is sound need to focus on enhancing intrinsic yield potential (Specht et al.^1999^; Vogel et al. 2021) rather than predominant defensive traits (stress tolerance, pest resistance etc.). Weather changes are expected to further adversely affect the major crop yields across the world, and will pose a key challenge to enhance yield and productivity through crop improvement in all crop species (Van Dijk et al. 2021a, b; Lesk et al. 2021; Hamed et al. 2025). A recent study about weather impact on crop yield suggested a compensatory effect of elevated CO_2_ on the yield of C3 crops like soybean, wheat, and rice; however, it can be outweighed by climatic stresses such as drought and heat (Rezaeiet al. 2023). It has been shown that under intensive drought conditions, elevated CO_2_ fails to enhance photosynthesis and yield in soybean due to altered stomatal function and canopy carbon balance (Gray et al. 2016).

Estimates suggest that photosynthetic efficiency (PE) in soybean remains well below its theoretical maximum, indicating substantial scope to improve radiation use efficiency (RUE) and canopy carbon assimilation to accelerate future yield gains (Zhu et al. 2010; Croceet al. 2024). Quantitative benchmarks highlight the magnitude of this gap. In soybean, RUE typically ranges from ~ 0.8 to 1.5 g biomass MJ⁻^1^ intercepted radiation under field conditions (Xu et al. 2021; Lopez et al. 2021), corresponding to an actual energy conversion efficiency of ~ 2.4% of total incident solar radiation- substantially below the theoretical C3 maximum of 4.6–5.1% (Zhu et al. 2008). Similar constraints exist in other C3 species. Wheat and rice achieve RUE values of ~ 2.2–2.8 gMJ⁻^1^ intercepted photosynthetically active radiation (PAR) (Kiniry et al. 1989; Moroyoqui-Parra et al. 2024), remaining far from their physiological limits. By contrast, C4 crop like maize operates at higher efficiency, with RUE commonly exceeding 3.5 g MJ⁻^1^ intercepted PAR in optimal conditions (Kiniry et al. 1989; Lindquist et al. 2005) and modern elite hybrids reaching ~ 1.5–2.0 g MJ⁻^1^ total solar radiation (Messina et al. 2022). Notable, long-term maize breeding has exploited improvements in RUE, with approximately ~ 32% of historical yield gains attributed to enhanced RUE, highlighting the unrealized opportunity for genetic improvement in soybean (Messina et al. 2022).

The maximum achievable yield by a crop, often called its yield potential (Y*p*), can be defined as the maximum amount of yield (seeds, fruits, or biomass) when the best crop variety is grown in optimized conditions without any stress (biotic or abiotic) (Evans and Fischer 1999). The key determinants of crop yield potential are light availability and capture efficiency, light energy conversion, and plant architecture. Among these determinants, energy conversion efficiency (***ε***_***c***_), often expressed as RUE, remains the major untapped component of yield potential in C3 crops, including cereals and legumes (Zhu et al. 2010; Long et al. 2015). This efficiency is ultimately determined by photosynthetic efficiency (Long et al. 2006). ***ε***_***c***_ is essentially the energy equivalent of RUE, which measures the amount of biomass produced per unit of intercepted radiation. Photosynthesis is the fundamental process in crop plants and natural vegetation. It converts light energy and atmospheric carbon dioxide into organic compounds, thus serving as a direct or indirect source of all food (Page and Parry 2025). Theoretically, crop yield is directly dependent on the cumulative effect of photosynthetically active radiation (PAR) absorbed by the crop during the growing season, which gets translated into final seed yield (Long et al. 2006; Edreira et al. 2020). Enhancement of the photosynthetic efficiency has often been reported as a strategy to increase seed yield in crop plants (Long et al. 2006; Simkin et al. 2019; Croce et al. 2024; Wu et al. 2019, 2023). Long-term maize breeding demonstrates the value of improving RUE, with approximately 32% of historical yield gains attributed to enhanced RUE despite little change in light interception (Messina et al. 2022).

In contrast, the inherently lower light conversion efficiency of C3 crops including soybean, makes improving PE as even greater breeding opportunity than in C4 crops such as maize. Soybean yield is determined by components such as pod number, seed number, and individual seed weight. Unlocking the genetic potential for improved PE holds significant promise for improving soybean seed yield potential (Köhler et al. 2017; Simkin et al. 2019). Recent modeling syntheses indicate that yield gains from improving photosynthesis are scenario dependent (Matthews and Burgess 2024). For soybean, a coupled BioCro-ePhotosynthesis framework predicted an average ~ 6.75% seed yield increase across four field seasons when phosphoglycerate kinase (PGK) and phosphoribulokinase (PRK), Calvin-Benson cycle enzymes were increased two-fold (He et al. 2024).

**Fig.1:**
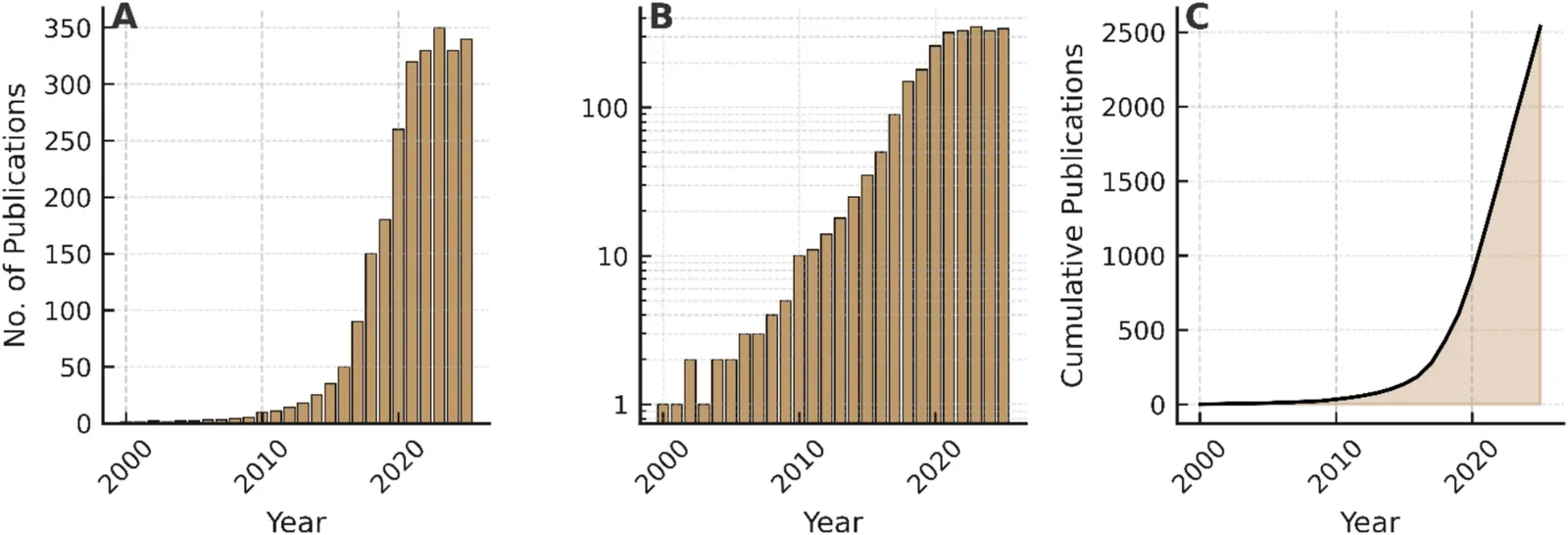
26-year trend of global scientific publications (2000–2025) based on PubMed database, including **a** articles with keywords “Crop”, “Photosynthesis”, and “Yield”, **b** “Soybean”, “Photosynthesis”, and “Yield”, and **c** cumulative publications on identical search parameters. Data collected in September 2025

There has been a significant increase in scientific research focused on the role of photosynthesis in crop yield improvement over the past 25 years. Notably, the number of publications on photosynthesis, yield, and improvement (Fig. 1a) have steadily grown, especially in recent years, indicating a growing interest in optimizing photosynthetic processes to enhance crop productivity. When narrowing down to research focusing on soybean (Fig. 1b), the upward trend is also evident, although the volume of publications is smaller. This reflects a targeted but still expanding focus on soybean, underscoring the recognition of photosynthesis as a critical factor for improving soybean seed yield (Croce et al. 2024; Garcia et al. 2023). As a globally important crop in terms of meal protein and oil production, there is an urgent need to improve soybean seed yield potential under resource-limited and increasingly variable climatic conditions (Faralli and Lawson 2020; Rezaei et al. 2023; Tran et al. 2025). Enhancing PE in soybean could offer a sustainable pathway to achieve higher seed yield without requiring proportional increases in agricultural inputs and resources such as arable land (Bailey-Serres et al. 2019). This research trend aligns with the goals of modern soybean breeding, where integrating photosynthetic traits into a cultivar development pipeline could provide a competitive advantage in meeting future food and feed demands while mitigating challenges posed by increasing environmental stressors.

Notably, the term PE has been applied with considerable flexibility in crop science literature, encompassing a wide range of traits. These traits vary from net carbon assimilation rate, radiation use efficiency, and maximum quantum efficiency of PSII to apparent photosynthesis and maximum rate of carboxylation (Slattery et al. 2017; Xu et al. 2021; Koester et al. 2016; Zhang et al. 2016; Montes et al. 2022). To ensure clarity in this review, we will collectively refer to these diverse components as ‘photosynthetic efficiency (PE)’, encompassing all properties that contribute to enhanced conversion RUE in the crop. In the present review, we explore the concept of PE and different factors—morphological, physiological, management, and environmental—that influence this efficiency, with a particular focus on soybean. We will examine the genetic basis of several important photosynthetic traits that hold potential for impacting the soybean yield components, as well as the extent to which these relationships are modulated by different breeding, environment, and management practices. Additionally, we will discuss the role of modern high-throughput phenotyping technologies and multi-omics for studying PE traits and their relationships with yield, comparing their utility in applied soybean breeding. Finally, we will explore future directions, emphasizing the integration of these complex PE traits into soybean breeding programs using machine learning and genotype × environment × management (G×E×M)-based predictive modeling.

## Photosynthesis and factors impacting photosynthetic efficiency

Photosynthesis is fundamental to life on Earth. Plants use sunlight and water to convert CO_2_ into biomolecules required for their growth and development (Page and Parry 2025). Photosynthesis originated about 2 billion years ago in an atmosphere rich in carbon dioxide (CO_2_) and lacking in oxygen (O_2_). This immensely important life process can be described in the form of a chemical equation as:

$$6{\mathrm{CO}}_{2} + 12{\mathrm{H}}_{2} {\mathrm{O}} + {\text{light energy}} \to {\mathrm{C}}_{6} {\mathrm{H}}_{12} {\mathrm{O}}_{6} + 6{\mathrm{O}}_{2} + 6{\mathrm{H}}_{2} {\mathrm{O}}$$

**Fig.2:**
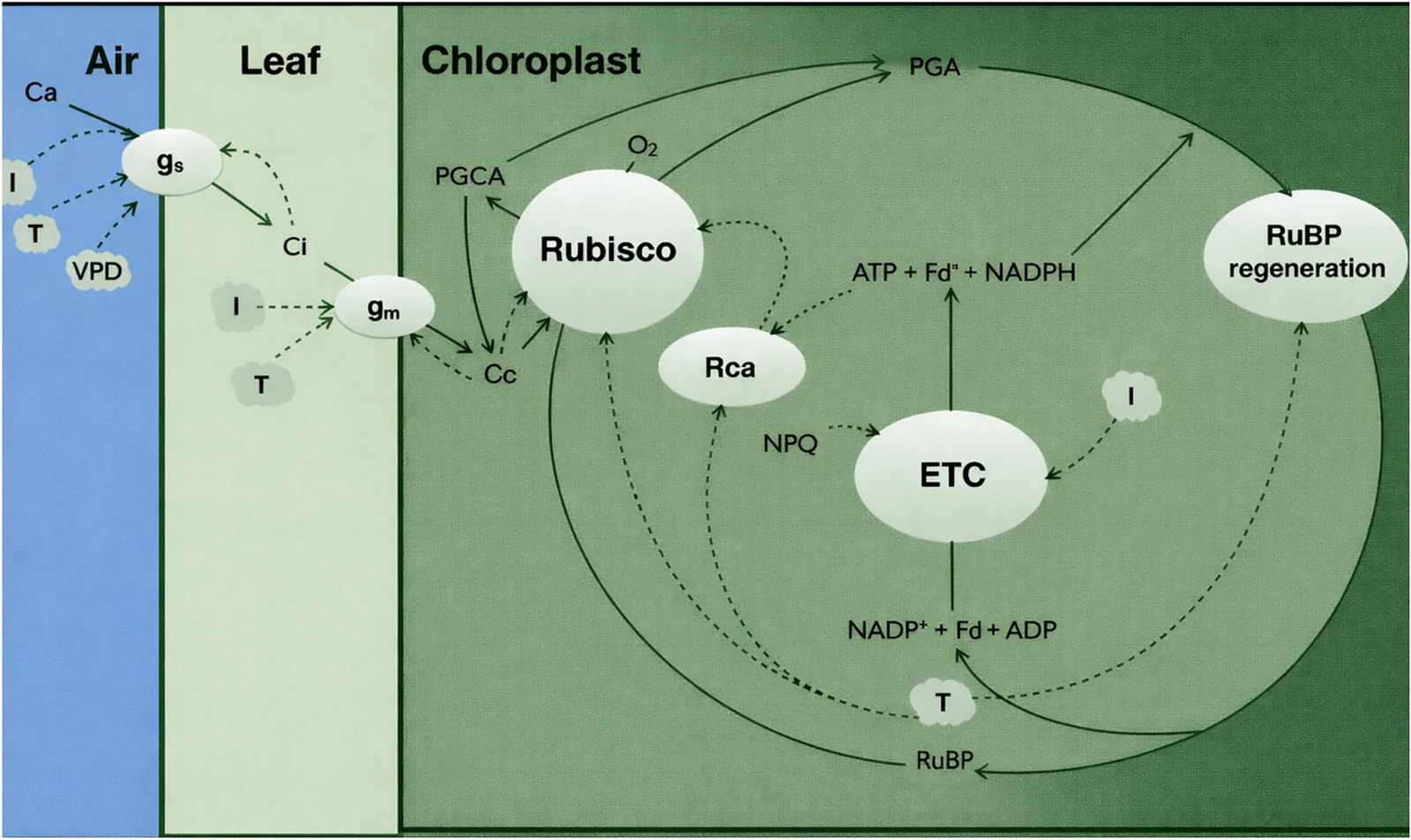
Major components and processes of dynamic photosynthesis and key environmental influences. CO_2_ is denoted as Ca (ambient), Ci (intercellular), and Cc (chloroplastic). Abbreviations: ETC, electron-transport chain; Fd, ferredoxin; gₘ, mesophyll conductance; gₛ, stomatal conductance; I, irradiance; NPQ, non-photochemical quenching; PGA, 3-phosphoglycerate; PGCA, 2-phosphoglycolate; Rca, Rubisco activase; Rubisco, ribulose−1,5-bisphosphate carboxylase/oxygenase; RuBP, ribulose−1,5-bisphosphate; T, temperature; VPD, vapor pressure deficit

Plants acquire energy from visible light to extract electrons from water molecules, utilizing them for converting atmospheric carbon dioxide (CO_2_) into biomass. Dynamic photosynthesis can be broken into three key phases: photosynthetic induction, post-illumination CO_2_ uptake, and the post-illumination CO₂ burst. Photosynthetic induction depends on processes such as RuBP regeneration, Rubisco activation, and stomatal opening. Changes in mesophyll conductance (gₘ) and non-photochemical quenching (NPQ) in response to changing light can also influence photosynthesis, and these responses are further shaped by CO_2_ levels and leaf temperature (Kaiser et al. 2015) (Fig. 2).

In the Calvin-Benson cycle (CBC), an enzyme called ribulose 1,5-bisphosphate carboxylase/oxygenase (Rubisco) plays a key role in fixing the atmospheric CO_2_. Naturally, it exhibits a higher affinity for CO_2_ than for O_2 _(Bowes et al. 1971), reflecting its evolution in an O_2_-limiting atmosphere. An alternative process called photorespiration begins after O_2_reacts with RuBP instead of CO_2_. It is also catalyzed by the Rubisco enzyme. Photorespiration produces one molecule each of 3-phosphoglycerate and phosphoglycolate. It takes a significant amount of energy (ATP and NADPH) for recycling these compounds. Photorespiration is essential in plants that facilitate photosynthesis in an oxygen-rich environment by breaking down 2-phosphoglycolic acid (2PG), an inhibitor of key enzymes in CBC. However, it significantly lowers the efficiency of photosynthesis in C3 plants via reduction in carbon assimilation, leading to about 20–40% yield penalties (Walker et al. 2016).

Yield improvement in major cereals over the past 55–60 years has resulted from the combined effects of genetic improvement and agronomic practices. Case studies in wheat and maize indicate that roughly 30–60% of long-term yield progress can be attributed to genetic gain, with the remainder explained by changes in fertilizer use, plant density, pest control, irrigation, and mechanization (Bell et al. 1995; Duvick 1984; Smith et al. 2014).

In soybean, retrospective analyses using historical cultivar series and on-farm data clearly demonstrate measurable genetic gains in seed yield over time, but also show that these gains cannot fully explain observed yield trends (Koester, 2014; Krause et al. 2023). For example, Rowntree et al. (2013) estimated that roughly half of the long-term yield increase in US soybean was attributable to improved agronomic practices, such as earlier planting and optimized maturity selection, with the remainder explained by genetic improvement. Together, these studies support a balanced interpretation: soybean breeding has delivered important genetic advances, but a substantial share of realized yield improvement at the farm level has depended on agronomic and mechanization gains that allow varieties to realize their genetic potential (Rowntree et al. 2013; Suhre et al. 2014; Mourtziniset al. 2017, 2021; Grassiniet et al. 2015).

Parallel advances in soil fertility and nutrient management, integrated weed and pest control, and widespread adoption of conservation tillage and high-efficiency mechanization have further enhanced resource capture and stress avoidance (Grassini et al. 2015; Córdova et al. 2020; Di Mauroet al. 2022). Physiological analyses of historical soybean varieties series indicate that modern varieties achieve higher yield potential through changes in canopy architecture, radiation use efficiency, and assimilate partitioning; however, water temperature and management-related stresses remain dominant constraints on fully realizing this potential under field conditions (Koester et al. 2014; Vogel et al. 2021).

The green revolution in wheat and rice is a prime example of this genotype × management synergy, where semi-dwarf, input-responsive varieties enable massive yield boosts when deployed alongside irrigation, fertilizers, and modern farming technologies(Liu et al. 2022; Reynolds et al. 2009; Crespo-Herrera et al. 2017). Despite these historical gains, the rate of yield improvement in several major crops has slowed in recent decades, and farm yields in many regions remain well below biophysical yield potential (Gerber et al. 2024). Against this backdrop, there is growing interest in enhancing intrinsic yield potential, particularly through improvements in PE and RUE, as a pathway to further enhance soybean productivity under future weather scenarios.

Considering the continuous shrinking of arable land and environmental challenges, it is paramount to explore novel pathways to boost crop yields. Improving crop yield potential (*Yp*) is one of the promising approaches to achieve this objective. Splitting yield potential into major components enables assessment of targeted multiplicative improvements. *Yp* is the product of several underlying sub-components:

$$Y_{p} = Q \times {\epsilon}_{i} \times {\epsilon}_{c} \times {\epsilon}_{p}$$

where Q is the total incident solar radiation (MJ/m^2^) throughout the growing season; ***ε***_***i***_ is the efficiency with which the plant intercepts/absorbs solar radiation; ***ε***_***c***_ is the efficiency with which the intercepted radiation is converted into biomass (also called radiation use efficiency or RUE); ***ε***_***p***_ is the partitioning efficiency (i.e., harvest index), which is the proportion of biomass allocated to harvested product (Long et al. 2015). The energy conversion efficiency of C3 crops is 2.4% (with a theoretical maximum of 4.6–5.1%), and for C4 crops, it is 3.7% (with a theoretical maximum of 6%), based on the total incident solar radiation intercepted by the leaf canopy (Zhu et al. 2008). In soybean (*Glycine max* (L.) Merr.), ***ε***_***i***_ reaches values upto0.9, which is considered highly optimized (Zhu et al. 2010; Koester et al. 2016) along with ***ε***_***p***_***,*** thus suggesting substantial potential in improving RUE.

Despite its central role in determining crop productivity (Lawson et al.^2012^; Garcia et al. 2023), PE has historically been difficult to incorporate into breeding programs. This limitation has largely resulted from the complexity of the photosynthetic processes, which involves numerous interacting metabolic pathways and environmental responses (Stirbetet al.^2024^; Croce et al. 2024), as well as from the lack of reliable high-throughput field phenotyping methods to quantify these traits. Recent advances in high-throughput phenotyping and remote sensing technologies are beginning to alleviate this constraint, creating new opportunities to systematically evaluate photosynthetic traits and exploit their genetic variation for yield improvement in soybean.

Morphological traits contributing to PE span a wide range of scales, from cellular to canopy levels. At the leaf anatomical level, traits such as leaf thickness, chloroplast distribution, stomatal density, mesophyll cell structure, and leaf hairs have been shown to significantly impact PE. For instance, leaf thickness influences light absorption, with thicker leaves intercepting more light (Sinclair et al. 1971; Ustin and Jacquemoud 2020). Physiological traits such as stomatal regulation, Rubisco activase dynamics, and biomass synthesis play crucial roles in PE. Stomatal regulation, which controls the exchange of gases between leaves and the atmosphere, can influence PE by balancing water loss and CO_2_ uptake (Liu et al. 2005; Sharkey et al. 2001). Rubisco activase, an enzyme essential for the catalytic activity of Rubisco, can limit photosynthetic rates under certain environmental conditions, such as low or high temperatures and varyingCO_2_ concentrations (Sharkeyet al. 2001). Biomass synthesis, while essential for growth and development, can also constrain PE, especially when excessive biomass accumulation occurs (Gotoh et al. 2018). Optimizing the interplay between these anatomical and physiological traits is key to enhancing PE. However, current research has limitations in fully understanding the optimal combinations of these traits and their interactions under complex agricultural environments. Therefore, the primary factors impacting PE in C3 crops, especially legumes, can be categorized into:

### Leaf anatomical and morphological traits

At the leaf scale, variation in anatomical and biochemical traits directly determines the biochemical capacity for carbon fixation. In soybean, stomatal density varies from 93 ± 3 to 166 ± 4 mm⁻^2^ across diverse accessions, and this variation is associated with differences in stomatal conductance (g_s_) and light-saturated photosynthetic rate (A_sat) (Sakoda et al. 2019). Modern elite varieties exhibit up to 23% greater daily photosynthesis than historical cultivars, attributed in part to increased g_s_ under high soil moisture (Koester et al. 2016). However, photorespiration imposes a substantial carbon cost in soybean, with theoretical yield penalties estimated at 20–40% under current atmospheric O_2_/CO_2_ ratios (Walker et al. 2016).

*Stomatal conductance*(g_s_): Stomata anatomical traits, like conductance, play an important role in photosynthesis and transpiration. It determines the gaseous exchange between the leaf interior and the external environment. Response of stomatal conductance has recently been shown to play an important role in photosynthetic induction under fluctuating light conditions in C3 crops like tomato (Yoshiyama et al. 2024). They found that plants with smaller stomata at higher density had rapid photosynthetic response compared to the opposite phenotype, indicating the usefulness of anatomical features affecting PE.

*CO*_*2*_* fixation activity* (*carboxylation efficiency*): Another approach to enhance photosynthesis is by increasing the biochemical capacity for CO_2_ assimilation, specifically by improving the carboxylation efficiency of Rubisco in C3 crops such as soybean (Flexas et al. 2016). Enhancing the RuBP carboxylation by Rubisco can potentially improve water-use efficiency (WUE) by reducing CO_2_ concentration needed to maintain higher photosynthetic rates (Carmo-Silva et al. 2015). Boosting RuBP regeneration has also been reported to enhance photosynthesis in C3 plants (Lefebvre et al. 2005; Rosenthal et al. 2011).

*Photosynthetic induction*: When a leaf moves to full light after a period of shade in the field, its CO_2_ assimilation rate does not instantly go up to maximum but progresses gradually in several minutes to become stable. This process is called photosynthetic induction, which significantly lowers the efficiency of CO_2_ fixation (Taylor and Long 2017; Soleh et al. 2017). The delay in Rubisco activation is considered a key limitation to speed of photo-induction, others being stomatal opening and mesophyll conductance (Sakoda et al. 2020). So, improving the speed of Rubisco activation can be beneficial at the canopy level to optimize photosynthesis in fluctuating lighting, further enhancing crop productivity in C3 crop plants like soybean.

*Non-photochemical quenching (NPQ):* Leaves dissipate the excess light energy in form of heat when they receive more energy in full sunlight, termed as non-photochemical quenching. It is quite an important phenomenon to reduce the production of oxidizing radicals, which would damage the photosynthetic apparatus (PSII) of leaves. Due to the relaxing mechanism of NPQ after coming back to optimal energy level, PE also gets compromised further. Recently, transgenic studies have shown the ability to enhance PE and biomass production in soybean, tobacco, and rice through the introduction of more rapid changes in response to fluctuating light conditions (De Souza et al. 2022; Kromdijk et al. 2016; Hubbart et al. 2018).

*Mesophyll conductance*: It plays a crucial role in photosynthesis by facilitating the movement of carbon dioxide from intercellular air spaces to sites of carbon fixation within the chloroplasts. Increased mesophyll conductance can make more CO_2_ available for plants to turn into food (sugars). This phenomenon has been explored in some other plants, including tobacco, through alteration of cell wall properties, leading to improved mesophyll conductance and photosynthesis (Salesse-Smithet al. 2024).

*Leaf characters*: Some field studies involving chlorophyll-deficient soybean mutants have demonstrated a significant reduction in leaf mass accumulation and seed yield (Slattery et al. 2017; Sakowska et al. 2018; Genesio et al. 2020). These findings highlight the complexity of this trait, which is affected by factors such as genes/pathways involved, degree of chlorophyll reduction, and canopy architecture under dynamic environmental conditions.

*Chloroplast size*: Manipulating chloroplast size in tobacco did not result in higher PE in a recent study by Głowacka et al. (2023). A similar outcome can be expected for other C3 crops like soybean. However, it has been demonstrated that under heat stress, variations in chloroplast size influence net photosynthesis in different soybean varieties (Herritt and Fritschi 2020).

*Electron transport chain rate (ETCR)*: ETCR significantly affects PE in inverted soybean leaves, especially under high temperature and high light conditions (Wang et al. 2022a). Studies have also shown that higher CO_2_ concentrations in soybean leaves lead to an increase in the maximum rate of electron transport and electron use efficiency, ultimately enhancing photosynthesis (Drag et al. 2020).

*Photorespiration*: Despite its essential role in plant metabolism in an O_2_-rich environment, adverse effects of photorespiration on PE have been evident. Consequently, a massive yield reduction in soybean of 36% has been reported, attributable to photorespiration (Walker et al. 2016). There have been several efforts to mitigate the oxygenation of Rubisco, such as bypassing photorespiration by alternative pathways, converting 2PG into CO_2_ or other intermediates of the Calvin cycle (Smith et al. 2023). Environmental conditions make this interaction confounding by reducing the affinity of Rubisco to CO_2_ with a rise in temperature compared to O_2_, enhancing the rate of oxygenation. However, the effect is opposite as the concentration of CO_2_ increases (Walker et al. 2016).

### Canopy structural traits

Canopy structure determines how efficiently intercepted radiation is distributed across photosynthetic surfaces/organs. In modern soybean, leaf area index frequently exceeds 4–6, leading to a self-shading lower canopy leaves. These leaves receive < 10% of incident photosynthetically active radiation and operate below light-compensation point for most of the day (Srinivasan et al. 2017; Soares et al. 2021). Because C3 leaf photosynthesis typically saturates at approximately 25% of full sunlight, upper canopy light saturation and lower canopy light limitation represent a primary structural bottleneck to radiation use efficiency (RUE) (Zhu et al. 2012). Recent evidence indicates that narrower, more erect leaf shapes can reduce upper canopy saturation and improve light penetration, supporting a 5–10% potential gain in canopy photosynthesis through architectural optimization (Tamang et al. 2026; Sreekanta et al. 2024).

*Canopy architecture*: Canopy photosynthesis represents the cumulative photosynthetic CO_2_ assimilation rate of all leaves within a canopy. The top leaves typically experience higher light intensity compared to the lower layers (Zhu et al. 2012). Leaf orientation and canopy architecture can influence PE by affecting light interception and distribution (Niinemets 2007). Canopy photosynthesis is regulated by both the microclimatic factors within the canopy (CO_2_, light intensity, temperature, and humidity) as well as the photosynthetic profiles of all the leaves within the canopy. The smart canopy concept has been put forward to optimize photosynthetic performance in crops. It involves absorption of far-red photons, gradually enhancing their interception in the lower part of the canopy (Ort et al. 2015). It is proposed to facilitate better use of incoming light, as leaves of the upper canopy, typically exposed to full sunlight, would have no benefit in absorbing far-red light.

*Leaf area index (LAI)*: LAI directly affects light interception and distribution within the plant canopy. Higher LAI typically means more leaf area available for capturing sunlight, crucial for photosynthesis. However, this relationship is complex, as it not only enhances light absorption but can also lead to shading of lower canopy leaves, which may not receive sufficient light to achieve optimal photosynthetic rates. LAI is shaped by both genetic and environmental factors, with leaf morphology (size, thickness, and arrangement) strongly influencing resource allocation and PE(Villar et al. 2021). While modern soybean varieties often produce excessive leaf area (Srinivasan et al. 2017; Soares et al. 2021), alternative canopy structures, including narrower and more erect (steeper leaf angle) leaves, can reduce upper-canopy light saturation, and improve light penetration and nitrogen use efficiency. It will ultimately support more effective canopy photosynthesis (Tamang et al. 2026).

*Non-foliar photosynthesis (NFPh)*:NFPh in soybean, such as chlorophyll-deficient mutants, can impact PE by altering light absorption and distribution within the canopy. These mutants exhibit slower NPQ, which has been shown to reduce biomass accumulation due to decreased photosynthesis (Acebron et al. 2023).

### Environmental variables

Environmental modulation of leaf-level biochemistry and canopy microclimate introduce substantial variation in realized PE. In soybean, optimal leaf photosynthesis occurs between 25–30 °C, whereas temperature above 35 °C reduce Rubisco activase activity and PSII photochemical efficiency, increasing photorespiratory carbon loss (Sharkey et al. 2001; Herritt and Fritschi 2020). Varying light intensities can lead to differences in PE. Higher light intensities can enhance photosynthesis up to a certain saturation point, beyond which plants may face oxidative damage to light-harvesting complexes (LHCs). This condition can trigger photoprotective mechanisms, reducing the quantum yield of PSII and daily carbon uptake of the plant. Additionally, shade-tolerant soybean varieties show better acclimation to light changes and higher biomass production compared to shade-intolerant varieties (Su et al. 2023). Water deficit limits g_s_ and intercellular CO_2_ concentration, reducing net assimilation before biochemical capacity is directly impaired (Silva-Pérez et al. 2020). Although elevated atmospheric CO_2_ can partially offset these limitations by suppressing Rubisco oxygenation, this compensatory effect is eliminated under intensive drought because altered stomatal function restricts canopy carbon balance (Gray et al. 2016).

### Agronomic Management practices

Management practices modify the soil-plant-atmosphere continuum by altering nutrient availability, soil physical properties, canopy structure and biotic interactions, thereby influencing the expression of photosynthetic traits. Nutrient management is particularly important because nitrogen deficiency reduces chlorophyll content, Rubsico activity and CO₂ assimilation, leading to leaf yellowing and lower PE (Qiang et al. 2023). Interestingly, fluctuating light can partially alleviate this limitation by accelerating photosynthetic recovery in N-deficient leaves (Li et al. 2020). Likewise, phosphorus application enhances chlorophyll content, net photosynthesis and light use efficiency under shaded conditions (Zhao et al. 2022), while organic amendments such as biochar and organic fertilizers improve chlorophyll content, g_s_, CO_2_ assimilation, WUE, and leaf area index (Wu et al. 2024; Dossin et al. 2024).

Soil and crop management practices also regulate canopy photosynthesis by improving resource capture and reducing environmental stress. Cover crop increased drought season WUE by 30% and V_cmax_ by up to 42%, highlighting their role in sustaining PE under limited water (Whippo et al. 2024). Similarly, conventional or reduced tillage systems, as well as tillage combined with film mulching, improve soil moisture and temperature, resulting in greater LAI, net photosynthetic rate and g_s_ than no-tillage systems (Buczek et al. 2022; Wang et al. 2022b; Wu et al. 2025). Plant density and row spacing further influence canopy light distribution- narrower rows accelerate canopy closure and greater light interception, whereas excessive density increases self-shading and can reduce RUE if LAI exceeds the optimal range for a given genotype.

Agrochemicals such as herbicides and pesticides also have a detrimental effect on the photosynthetic process in crops. Studies have shown significant adverse effects of glyphosate on photosynthesis, nutrient accumulation, and nodulation in non-GM (glyphosate-sensitive) soybean varieties(Zobiole et al. 2012; Krenchinski et al. 2017). Likewise, weed interference suppresses PE through resource competition and shade avoidance responses by altering light signaling and photosynthesis-related gene expression (Horvath et al. 2015). Early-emerging and highly competitive weeds such as common ragweed (*Ambrosia artemisiifolia*) and barnyardgrass (*Echinochloa crus-galli*), when present with significant densities at crop emergence, can strongly suppress soybean photosynthesis and growth through prolonged competition for light and resources (Swanton et al. 2015; Millar et al. 2011).

### Root system architecture and below ground interactions

Soybean is a legume that meets a substantial portion of its nitrogen demand through symbiotic nitrogen fixation. Root system architecture (RSA) traits, including primary root length, lateral root number and convex hull area, determine nodule distribution and nitrogen acquisition capacity. Because nitrogen is a major constituent of Rubisco and chlorophyll, RSA indirectly constrains leaf level biochemical capacity for carbon fixation. Nodulated roots also act as significant carbon sinks. The strength of this sink can stimulate leaf photosynthetic rates to meet carbon demand (Kaschuk et al. 2009). Recent genome-wide association studies in soybean have identified significant loci underlying RSA variation, providing genetic targets for improving below ground resource capture (Rathore et al. 2024) and comparative evidence indicates that RCA is genetically tractable across multiple crops (Zheng et al. 2020). Beyond nitrogen, root depth and density determine water uptake under drought conditions. Deep or densely branched root systems sustain stomatal conductance and photosynthetic rates when soil moisture is limited. Root derived hormonal signals, such as abscisic acid and cytokinins, further modulate stomatal behavior and leaf senescence, directly influencing canopy-level PE. Despite these connections, root phenotyping remains technically challenging in field settings. Also, the genetic correlation between RSA and shoot photosynthetic traits in soybean remains to be further quantified (Wang et al. 2022a, b, c). Integrating below ground phenotyping with aboveground HTP is therefore an emerging priority for photosynthesis-informed breeding.

Despite the multiple factors influencing PE, four interconnected bottlenecks constrain soybean yield potential practically. First, photorespiration imposes a quantified yield penalty of ~ 36% under current atmospheric CO_2_ (Walker et al. 2016). It diverts carbon and energy away from assimilate accumulation and thereby limiting both pod number and individual seed weight (Bruening and Egli 1999). Second, suboptimal light distribution within the canopy- exacerbated by excessive leaf area in modern soybean cultivars (Srinivasan et al. 2017; Soares et al. 2021) suppresses lower canopy photosynthesis including pod photosynthesis. Insufficient light penetration to the lower canopy during flowering has been linked to reduced pod number per unit area (Kantolic et al. 2013; Feng et al. 2019), while experimental pod shading during seed filling reducing individual seed weight by 13–14% (Cho et al. 2023). This inefficiency limits RUE to ~ 0.8–1.5 g biomass MJ⁻^1^ intercepted radiation (Xu et al. 2021; Lopez et al. 2021), well below the theoretical C3 maximum energy conversion efficiency of 4.6–5.1% (Zhu et al. 2008). Third, the historical lack of high-throughput field phenotyping has prevented systematic selection for PE traits that underpin yield components. Chlorophyll content, a proxy for PE, correlates with seed yield at r = 0.26–0.90 across multiple studies yet its low-throughput measurement has limited breeder access to this variation for improving pod number, seed weight, and harvest index (Theeuwen et al. 2022). Finally, the narrow genetic base of elite soybean germplasm (Hyten et al. 2006; Mikel et al. 2010; Kofsky et al. 2018) limits allelic diversity for RUE-related traits. Because historical breeding has already optimized light interception (εi ~ 0.9; Zhu et al. 2010; Koester et al. 2016) and partitioning efficiency (HI ~ 0.35–0.45; Jiang et al. 2019; Naydenova et al. 2022), further gains in pod number, seed weight, and overall yield depend on introducing novel alleles for canopy carbon assimilation and RUE. Together, these bottlenecks indicate that improving RUE and canopy carbon assimilation offers the most direct physiological route to increase soybean yield components and ultimately, harvest index.

## Field phenotyping of photosynthetic traits: tools, scales, and breeding relevance

Emerging phenotyping technologies are transforming breeding programs by enabling more precise and timely selection of high-yielding, stress-resilient genotypes from larger populations, ultimately accelerating genetic gain in cultivar development pipelines (Alves et al. 2024; Jones et al. 2024; Araus et al. 2018; Santana et al. 2022). Traditional selection criteria in soybean breeding, such as lodging resistance, canopy architecture, and seed quality traits, are typically assessed at physiological maturity and often overlook the physiological dynamics throughout the growing season. These seasonal changes, particularly in photosynthetic efficiency (PE), are associated with final crop yield potential and stress resilience (Muhie 2022; Araus et al. 2021; Elsalahyand Reckling 2022).

To address this gap, a range of phenotyping tools and techniques has been developed, allowing breeders to quantify PE at different spatial scales, canopy positions, and growth stages. These approaches detailed leaf-level measurements using handheld instruments to canopy, canopy-scale assessments obtained from ground-based systems and aerial remote sensing technologies (Song and Zhu 2024; Taylor 2024).

Field phenotyping platforms for PE operate at three distinct spatial scales, each with specific advantages and limitations for soybean biology. Handheld devices (pulse-amplitude-modulated fluorometers, SPAD meters, portable gas exchange systems) provide direct mechanistic measurements of leaf-level parameters such as A_sat_, g_s_ and Fv/Fm with high accuracy. However, they are inherently low throughput, labor intensive and poorly suited for dense soybean canopies after canopy closure, particularly in indeterminate genotypes that continue stem elongation through reproductive development (Borra-Serrano et al. 2020). At the plot scale, ground-based phenotyping vehicles (mobile rovers, buggies, fixed gantries) can carry heavier sensor payloads including LiDAR, thermal cameras, and hyperspectral spectrometers while maintaining close sensor-canopy proximity for high-resolution measurements (Deery et al. 2014). These systems are particularly effective for quantifying canopy height, leaf angle and 3D architecture that are difficult to capture from aerial views. Their limitation in soybean is field accessibility as lodging, wet soil and narrow row spacing can restrict movements. At the largest field scales, unmanned aerial vehicle (UAVs) equipped with multispectral, hyperspectral or thermal sensors enable high-throughput assessment of hundreds of plots within a single flight, supporting repeated temporal monitoring throughout the season (Alves et al. 2024; Borra-Serrano et al. 2020). UAVs are especially valuable during the reproductive stages (R2–R6), when vegetation indices shows the strongest relationships with soybean agronomic traits like yield components (Magar et al. 2025; Ranđelović et al. 2026). However, nadir view aerial imaging loses sensitivity once soybean approaches near complete canopy closure (ε_i_ ≈ 0.9), limiting the detection of lower canopy and pod photosynthesis in modern broad leaf varieties. Consequently, integration with ground-based LiDAR or oblique angle imaging is often required to resolve architectural traits variation below the upper canopy (Srinivasan et al. 2017; Tamang et al. 2026).

Although handheld gas exchange systems remain indispensable for mechanistic studies, there inherently low throughput makes them impractical for screening the screening the hundreds to thousands of genotypes typically evaluated across environments, seasons and management conditions in breeding programs (Theeuwenet al. 2022). Consequently, several high-throughput approaches have been developed to complement direct physiological measurements, including small handheld and aerial systems (Visakh et al. 2024; Song and Zhu 2024). Another benefit of these technologies is their ability to repeatedly phenotype the fields throughout the growing season rather than relying on a single time-point measurement. MultispeQ (PhotosynQ, USA), which combines a fluorometer and spectrometer enable rapid estimation of multiple chlorophyll fluorescence traits with environmental parameters (Kuhlgertet al. 2016). Similarly, the light-induced fluorescence transient (LIFT) system measures the canopy reflectance spectrum, and provides estimates of chlorophyll content, pigment composition, leaf nitrogen, and leaf photosynthetic parameters (Kelleret al. 2024; Song and Zhu 2024).

Unlike gas exchange systems, remote sensing platforms do not measure photosynthesis directly, instead they capture proxies correlated with underlying physiological processes. Various remote sensing-based platforms are being explored in legumes for high-throughput phenotyping, including multispectral, hyperspectral and thermal infrared imaging systems deployed fromUAVs or ground-based systems (Song and Zhu 2024; Santana et al. 2022). The choice among these technologies depends on the target trait, the genetic diversity of the population and the breeding or management objective.

Multispectral imaging captures reflectance in limited number of broad spectral bands (typically blue, green, red, red-edge, near-infrared). It is cost-effective approach to reliably estimate leaf area index, canopy cover and general greenness. Consequently, it is well suited for large-scale breeding trials, early-generation screening and monitoring management practices such nitrogen application and irrigation scheduling (Alves et al. 2024). However, its relatively limited spectral resolution limits detection of subtle biochemical variation associated with PE. Hyperspectral imaging overcomes this limitation by measuring reflectance across hundreds of narrow contiguous wavelengths (typically 400–2500 nm), enabling precise estimation of chlorophyll and carotenoid content, water status and photochemical reflectance index (PRI), all of which are mechanistically linked to leaf photosynthetic performance(Gamon et al. 1992; Meacham-Hensold et al. 2019). In soybean breeding populations, hyperspectral traits show moderate genetic correlations with V_cmax25_ and J_max25_ (r ~ 0.4–0.6; Montes et al. 2022), making them particularly valuable for genomic prediction and genetic mapping of complex physiological traits. Thermal infrared imaging provides an indirect estimate of stomatal conductance by measuring canopy temperature. Cooler canopies generally indicate higher transpiration rate and greater gs (Toum et al. 2022; Pignon et al. 2021), making thermal imaging especially useful for drought screening and evaluating management practices that influence plant water status, including irrigation scheduling and residue retention (Crusiol et al. 2020). Although thermal imaging has been more widely adopted in cereals, its application in soybean breeding remains comparatively limited and represents a promising area for future high-throughput phenotyping.

Thermal imaging helps researchers probe the temperature-based response at plant and canopy levels, which can convey insightful physiological signals induced by biotic and abiotic stressors such as water extremes, heat, soil salinity, or various pathogens and invasive species(Bianet al. 2019; Wen et al. 2023). Canopy temperature is significantly correlated with transpiration rate in soybean (Toumet al. 2022), implying the usefulness of thermal imaging for capturing useful photosynthetic variation. So, thermal imaging has been explored to dissect the natural variation in several photosynthetic traits such as stomatal conductance (Pignon et al. 2021), transpiration rate, leaf area index (Crusiol et al.^2020^), and leaf water potential (Cohen et al. 2005). These traits are shown to have a significantly large influence on PE at the canopy level. Until now, the usage of this technology has been mostly restricted to C4 crops such as sorghum and maize; however, it has the potential to revolutionize high-throughput photosynthetic phenotyping in C3 legumes, specifically soybean.

Hyperspectral imaging can be viewed as the phenomic equivalent of genome-wide prediction. Whereas multispectral imaging samples a limited number of spectral bands, hyperspectral imaging captures dense phenotypic information across the electromagnetic spectrum. It enables more comprehensive characterization of complex physiological traits. When combined with genomic prediction, this dense phenotypic representation is particularly valuable for improving PE in genetically diverse soybean germplasm. Numerous studies have demonstrated that hyperspectral and multispectral vegetation indices can estimate chlorophyll and carotenoid pigment content (Gamon et al. 1992; Gao et al. 2024; Wong et al. 2019), water stress responses (Crusiolet al. 2023; Kovar et al. 2019; Shi et al. 2024), and nutrient deficiencies (Debnath et al. 2021; Furlanettoet al. 2024), all of which strongly influence PE (Gu et al. 2024; Yassue et al. 2023).

Large-scale collection of spectral information also facilitates the identification of germplasm possessing superior photosynthetic performance. For example, chlorophyll content is consistently associated with V_cmax25_ and J_max25_. In the SoyNAM population (~ 5600 lines across two environments), moderate genetic correlations (r ≈ 0.4–0.6) were observed among chlorophyll content, V_cmax25_ and J_max25_ (Montes et al. 2022). Similar positive relationship between chlorophyll content and net photosynthetic rate (Pn) have been reported under diverse field environments (Croft et al. 2017; Jumrani et al. 2017; Sakowska et al. 2018). Spectral indices derived from multispectral and hyperspectral platforms have been shown to reliably estimate chlorophyll content and related traits across large populations in a rapid and non-destructive manner (Camino et al. 2018, 2019; Meacham-Hensold et al. 2019; Silva-Perez et al. 2018; Vilfan et al. 2019).

For remote sensing proxies to support PE-driven breeding, a mechanistic link must exist between the measured signal, the underlying physiological process and the yield component ultimately affected. The principal relationships in soybean are summarized as follows:

- For canopy structure and light interception (LiDAR, RGB photogrammetry, multispectral canopy cover)- UAV derived canopy height, volume and LAI serve as proxy for light interception efficiency (εi) and canopy architecture. Narrow, erect leaves improve light penetration to lower canopy layers, enhancing canopy photosynthesis (CAP) during later reproductive stages. Because pod and seed photosynthesis contribute approximately 9% of total canopy carbon gain and offset upto 81% of respiratory losses, improved architectural light distribution directly supports seedweight (Cho et al. 2023; Sreekanta et al. 2024).
- For chlorophyll and biochemical capacity (hyperspectral and multispectral red-edge)- Spectral indices such as NDRE and hyperspectral chlorophyll indices estimate leaf chlorophyll content, which is consistently and positively associated with V_cmax25_ and J_max25 _(Montes et al. 2022; Croft et al. 2017). Higher chlorophyll content sustains carbon assimilation rates through the critical reproductive phase, supporting pod number and seed filling duration, which are the dominant determinant of final seed yield (Egli2013; Kantolic et al. 2013).
- For stomatal conductance and water-use efficiency (thermal infrared)- Canopy temperature depression (cooler canopies) indicates higher transpiration and g_s_. In soybean, thermal derived g_s_ estimates correlate well with photosynthetic rate and yield retention under variable moisture regimes (Crusiol et al. 2020; Pignon et al. 2021). Management practices that sustain lower canopy temperatures such as cover cropping or residue retention (mulching), therefore protect PE and seed yield under moisture stress (Whippo et al. 2024).
- For photoprotection and PSII efficiency (fluorescence imaging, PRI)- The photochemical reflectance index and chlorophyll fluorescence track xanthophyll cycle activity and non-photochemical quenching (NPQ). While transgenic acceleration of NPQ relaxation has been proposed to boost biomass, natural variation in NPQ is being explored and PE differences are seemingly more strongly linked to Rubisco activation dynamics (Wang et al. 2020b). Thus, PRI and fluorescence are currently useful as stress indicators and for monitoring dynamic light acclimation rather than as direct yield predictors.

These relationships demonstrate how vegetation indices derived from multispectral, hyperspectral, thermal and fluorescence-based sensing can serve as reliable proxies for photosynthetic efficiency. By enabling rapid, non-destructive phenotyping across large breeding populations, these technologies provide scalable tools for integrating physiological information into modern soybean breeding pipelines.

## Natural variation and genetic architecture of photosynthetic traits in soybean

Phenotypic variation in photosynthesis is well documented across various crop species, including common bean (Keller et al. 2024), soybean (Montes et al. 2022), maize (Álvarez-Iglesias et al. 2022), chickpea (Panget al. 2025), pigeonpea (Álvarez-Iglesias et al. 2022), and wheat (Faralli and Lawson 2020). This suggests a genetic component for these variations in photosynthetic traits. Understanding the genetic architecture of these traits is crucial for crop improvement. In soybean breeding, it is imperative to connect variations in photosynthetic traits to genetics to utilize this knowledge for developing varieties with enhanced yield potential. These traits include, but are not limited to A_sat_, the light and CO_2_-saturated maximum photosynthetic rate (A_max_), maximum carboxylation capacity (V_cmax_), and the maximum electron-transport rate (J_max_). Despite this knowledge, there has been limited research focusing on natural genetic diversity related to PE traits and their impact on soybean yield components in real field conditions (Hu et al. 2024; Wang et al. 2022a, 2020a; Herritt et al. 2018). This could primarily be attributed to the limited capacity to accurately phenotype photosynthesis in the dynamic field conditions and effective utilization of phenotyping techniques in a high-throughput manner (Song and Zhu 2024).

Koester et al. (2016) found that modern soybean varieties generally exhibited greater daily photosynthetic carbon assimilation upto 23% compared to older varieties, largely attributed to enhanced stomatal conductance during periods of high soil moisture. A study by Hao et al. (2012) evaluated a set of 168 landraces for chlorophyll content and chlorophyll fluorescence parameters, including Fv/Fm: maximum quantum yield of PSII primary photochemistry in dark-adapted state, ABS/RC: light energy absorbed per reaction center, ETo/ABS: φEo, Quantum yield for electron transport, ETo/TRo: WEo, probability that a trapped exciton moves an electron into electron-transport chain beyond QA^−^, and PI_ABS_: performance index on absorption basis. The study revealed extensive genetic variation for all the traits, with most variation in PI_ABS_ (1.23–6.02), and least variation in Fv/Fm (0.77–0.84). Notably, G**×**E significantly impacted PE, as relatively lower heritability was observed for all the traits (37.6–47.3%). Shamim et al. (2022) assessed the photosynthetic efficiency of 74 Japanese soybean mini-core collection lines (GmJMC) in the field by using the ‘MIC-100’ phenotyping system. Their analysis revealed four distinct clusters based on light-saturated photosynthesis (A_sat_). Specifically, the accessions in cluster 2 were found to exhibit higher PE, with GmJMC47 exhibiting the highest values for A_sat_, chlorophyll content, g_s_, stomatal density (SDs), electron-transport rate (ETR), and light use efficiency of photosystem II (PSII) (Fv'/Fm'), along with the lowest NPQ. This indicates that GmJMC47 has enhanced CO_2_ supply and highly efficient light-harvesting mechanisms. In another study, Hu et al. (2024) screened 219 soybean accessions for PE at two growth stages for chlorophyll fluorescence parameters (ABS/RC, Chl, ETo/ABS, ETo/TRo, Fv/Fm, and PIABS). They reported wider variation in heritability among traits ranging from 16.8% (chlorophyll content) to 90.8% (ABS/RC) at the reproductive growth stage R2(Fehr and Caviness 1977), based on two yield field data sets. However, heritability at the reproductive growth stage R6 was generally lower (15.6% for ETo/TRo to 34.6% for PIABS) across 4 years. Notably, gas exchange traits, measured exclusively at the R2 growth stage (over 3 years) in a controlled net house, showed moderate to higher heritability (50.3% for transpiration rate to 85.2% stomatal conductance), with stomatal conductance showing maximum variability. Collectively, these studies indicate that the genetic architecture of photosynthetic traits in soybean is strongly trait- and developmental stage-dependent, with heritability generally higher at earlier reproductive stages and declining toward later stages. This pattern likely reflects increasing environmental and physiological complexity as the season progresses, including cumulative exposure to temperature and water stress, changes in source-sink relationships, and the onset of leaf aging and senescence. As reproductive development advances, these factors amplify G**×**E interactions, thereby reducing the proportion of phenotypic variance attributable to genetic effects. Consequently, photosynthetic traits expressed early in the reproductive phase may better capture intrinsic genetic differences among genotypes, whereas later stage measurements may reflect genotype-specific stress responses that are more environmentally dependent. These observations highlight the importance of growth stage-specific phenotyping and suggest that the timing of photosynthetic traits measurement is a critical consideration for their effective use in soybean breeding.

Under controlled conditions, Sakoda et al. (2016) evaluated 34 genotypes exhibiting light-saturated CO_2_ assimilation rates ranging from 18.1 to 27.6 µmol/m^2^/s. Among these, two maturity group IV accessions- PI 603911 C (Jiori, North Korea) and PI 594409 A (Jin dou, China), exhibited notably higher PE. These varieties showed elevated CO_2_ fixation rates along with higher V_cmax_ and J_max_. Importantly, PI 603911 C maintained superior performance under field conditions, with CO_2_ assimilation rates reaching up to 34.8 µmol/m^2^/s (11% higher than reference check- Tachinagaha), likely due to its elevated Rubisco content in the leaves. Stomatal density presents another promising target for enhancing leaf photosynthesis. Sakoda et al. (2019) explored variability in stomatal density among 90 soybean accessions. Using a high-throughput phenotyping system, they captured significant variation in stomatal density (93 ± 3 to 166 ± 4 mm^−2^). Furthermore, variations in leaf photosynthetic rates, attributed to differences in stomatal and Rubisco-related characteristics, have been observed in field conditions (Sakoda et al. 2021). These findings underscore the biochemical capacity in determining genotypic variation in PE.

Efforts have been made to investigate PE diversity in different breeding populations, including elite lines, Recombinant inbred lines (RIL), and nested association mapping (NAM) populations. For instance, Montes et al. (2022) leveraged the SoyNAM population of ~ 5600 F_5_-derived RILs from 40 biparental NAM populations grown in two environments (NE12 irrigated and IL13 rainfed). Each family was developed from crossing a unique founder line with common high-yielding parent IA3023 (Dairyland DSR365 x Pioneer P9381). Leaf hyperspectral reflectance was collected on 4796 plots (3/4th of design) in NE12, and 6,400 (full design) in IL13. After marker and line quality control, genetic analyses was conducted on 5171 F_5_-derived lines (plus 40 founders).This study revealed significant genetic diversity in key photosynthetic traits, such as V_cmax_ and J_max_, with values reaching up to 80 and134 µmol/m^2^/s, respectively. Notably, several founder lines with elite and exotic backgrounds, such as LD02-4485, exceeded the high-yielding parent IA3023 in both traits, highlighting potential for genetic improvement through targeted selection. These findings strongly support the exploration of soybean germplasm as an effective approach to improve resource use efficiencies related to photosynthesis.

Several studies attempted to uncover the genetics of photosynthetic traits in soybean, including fluorescence-based parameters, gas exchange parameters, and canopy-level traits by utilizing genetically diverse accessions as well as other breeding populations (Table 1). In a recent a genome-wide association study (GWAS) involving 219 soybean accessions, Hu et al. (2024) identified two key candidate genes, *GmIWS1* (*Glyma.01G012500*) and *GmCDC48* (*Glyma.03G182800*), associated with photosynthetic traits. *GmIWS1*was identified through stable InDel associations across environments for chlorophyll fluorescence parameters (Chl, ABS/RC, ETo/ABS, ETo/TRo, Fv/Fm, PI_ABS_). Based on expression profiling and positional information, *Gm1WS1,* encoding a transcriptional elongation factor, was selected as the most likely candidate gene. Six haplotypes of *Gm1WS1* were identified, with one haplogroup (H-II) exhibiting significantly higher values for multiple fluorescence traits and numerically greater seed weight and yield. The second gene, *GmCDC48,* was identified within a significant InDel cluster associated with gas exchange traits (stomatal conductance-Cond and intercellular CO_2_ concentration-Ci). This gene, encoding a cell division cycle protein of the AAA-ATPase family, was selected based on tissue expression and homology of *OsCDC48*, a known regulator of chloroplast development in rice. Four haplotypes of *GmCDC48* were identified, with one group (H-II) showing lower Cond and Ci but higher seed yield. While these candidate genes were proposed based on GWAS and expression data, further functional validation remains necessary to confirm their roles in regulating PE and seed yield in soybean.

**Table 1:** A summary of key QTLs and putative candidate genes identified in soybean associated with different photosynthesis-related traits

Photo synthetic traits	Trait(s) studied	QTL/locus/marker	Chr	Approx. position (Mb)	Effect size (R^2^/PVE/LOD)	Heritability (%)	Population type and size	Environment (s)	Evidence/Validation status	Candidate gene(s)/function	References
Chlorophyll fluorescence	Chl; ABS/RC; ETo/ABS; ETo/TRo; Fv/Fm; PIABS	*GmIWS1*/*Glyma.01G012500*	1	NR	NR	31.54–90.82	219 accessions	2	Candidate locus (single study; multiple traits)	Chr1: *GmIWS1* (*Glyma.01G012500*) transcription elongation	Hu et al. (2024)
	Fⱼ; F₀; M₀	*MPL1*/ss715578706	1	Chr1: 2.19	NR	Fⱼ 29.5; F₀ 36.8; M₀ 23.4	189 MG IV accessions	4	Candidate locus (single study; detected in multiple environments/years)	Chr1: *Glyma.01G017400*: RNA-binding family protein (nucleotide binding); *Glyma.01G028900*: ATP-citrate lyase; Chl synthesis	Herritt et al. (2018)
	Fᵢ; Fⱼ; Fₘ	*MPL2*/ss715582243	2	Chr2: 4.19	NR	Fᵢ 22.8; Fⱼ 29.5; Fₘ 20.6		4	Candidate locus (single study; detected in multiple environments/years)	Chr2: *Glyma.02G036300*—ATP-citrate lyase; Chl synthesis; *Glyma.02G047600*—*FNR1*	
	Fᵥ/Fₘ; φDₒ; φPₒ	*MPL3*/ss715588144	4	Chr4: 45.02	NR	Fᵥ/Fₘ 23.3; φDₒ 4.6; φPₒ 4.6		4	Candidate locus (single study; detected in multiple environments/years)	NR	
	M₀; φEₒ; ψₒ; Vⱼ	*MPL4*/ss715598351	7	Chr7: 5.52	NR	M₀ 23.4; φEₒ 2.6; ψₒ 2.5; Vⱼ 16.3		4	Candidate locus (single study; detected in multiple environments/years)	Chr7: *Glyma.07G057200*: thylakoid rhodanese-like protein (*TROL*; protein stability); *Glyma.07G059600*: *NdhV* (protein stability)	
	φEₒ; PIABS; ψₒ; Vⱼ	*MPL5*/ss715602131	8	Chr8: 43.90	NR	φEₒ 2.6; PIABS 3.2; ψₒ 2.5; Vⱼ 16.3		4	Candidate locus (single study; detected in multiple environments/years)	NR	
	ABS/RC; DI₀/RC; M₀; φEₒ; Vⱼ	*MPL6*/ss715606550	10	Chr10: 37.82	NR	ABS/RC 6.2; DI₀/RC 4.7; M₀ 23.4; φEₒ 2.6; Vⱼ 16.3		4	Candidate locus (single study; detected in multiple environments/years)	Chr10: *Glyma.10G153100*: *PsbP*; PSII	
	ABS/RC; DI₀/RC; Fₘ/F₀; Fᵥ/Fₘ; Fᵥ/F₀; φDₒ; φPₒ; PIABS	*MPL7*/ss715611132, ss715611140, ss715611141, ss715611188, ss715611189	11	Chr11: 7.67–8.10	NR	ABS/RC 6.2; DI₀/RC 4.7; Fₘ/F₀ 20.7; Fᵥ/Fₘ 23.3; Fᵥ/F₀ 20.8; φDₒ 4.6; φPₒ 4.6; PIABS 3.2		4	Candidate locus (single study; detected in multiple environments/years)	*HCF136* (PSII assembly); *GC1* (chloroplast division); *FBA2* (carbon metabolism); ATP-synthase and PSII proteins (energy conversion)	
	ET₀/RC; Fₘ/F₀; Fᵥ/F₀; M₀; φEₒ; ψₒ; Vⱼ	*MPL8*/ss715613004, ss715613007, ss715613098, ss715613099, ss715613101, ss715613103	12	Chr12: 3.98–4.60	NR	ET₀/RC 0.4; Fₘ/F₀ 20.7; Fᵥ/F₀ 20.8; M₀ 23.4; φEₒ 2.6; ψₒ 2.5; Vⱼ 16.3		4	Candidate locus (single study; detected in multiple environments/years)	Chr12: *Glyma.12G044500*: CLP protease subunit 6 (chloroplast structure); *Glyma.12G061600*: Rubisco large subunit	
	Fᵢ; Fₘ; TR₀/RC	*MPL9*/ss715615442	13	Chr13: 33.65	NR	Fᵢ 22.8; Fₘ 20.6; TR₀/RC 4.7		4	Candidate locus (single study; detected in multiple environments/years)	Chr13: *Glyma.13G213500*—Chr13: *Glyma.13G213500*: deoxyxylulose−5-phosphate synthase; *Glyma.13G215400*: NAD(P)-binding Rossmann-fold protein; *Glyma.13G225000*: aldolase-type TIM-barrel protein	
	ET₀/RC; Fₘ/F₀; Fᵥ/F₀; φEₒ; ψₒ; Vⱼ	*MPL10*/ss715616214	13	Chr13: 40.60	NR	ET₀/RC 0.4; Fₘ/F₀ 20.7; Fᵥ/F₀ 20.8; φEₒ 2.6; ψₒ 2.5; Vⱼ 16.3		4	Candidate locus (single study; detected in multiple environments/years)	Chr13: *Glyma.13G299200*: PSII *Psb27*; *Glyma.13G302100*: PSII reaction-center *PSB29*; *Glyma.13G302900*	
	ET₀/RC; ψₒ; Vⱼ	*MPL11*/ss715622432	15	Chr15: 49.05	NR	ET₀/RC 0.4; ψₒ 2.5; Vⱼ 16.3		4	Candidate locus (single study; detected in multiple environments/years)	Chr15: *Glyma.15G253700*: PSII subunit R; *Glyma.15G262700*: PSII reaction-center protein T	
	Fᵢ; Fⱼ; TR₀/RC	*MPL12*/ss715627511	17	Chr17: 38.69	NR	Fᵢ 22.8; Fⱼ 29.5; TR₀/RC 4.7		4	Candidate locus (single study; detected in multiple environments/years)	Chr17: *Glyma.17G228700*: cytokinin-responsive GATA factor 1 (Chl synthesis)	
	ET₀/RC; Fᵢ; Fₘ; Fₘ/F₀; Fᵥ; Fᵥ/Fₘ; Fᵥ/F₀; φDₒ; φPₒ	*MPL13*/ss715635516, ss715635520, ss715635524, ss715635529, ss715635531, ss715635532	19	Chr19: 45.60–45.79	NR	ET₀/RC 0.4; Fᵢ 22.8; Fₘ 20.6; Fₘ/F₀ 20.7; Fᵥ 17.3; Fᵥ/Fₘ 23.3; Fᵥ/F₀ 20.8; φDₒ 4.6; φPₒ 4.6		4	Candidate locus (single study; detected in multiple environments/years)	Chr19: *Glyma.19G212800*—sucrose synthase 3 (starch/sucrose metabolism)	
	ETo/ABS; ETo/TRo	Fluorescence association near *Glyma17g16370*	17	NR	2.61–2.75	37.6–38.3	168 landraces	NR	Candidate locus (single study)	Chr17: *Glyma17g16370*: tetraspanin-family membrane protein	Hao et al. (2012)
	Fv/Fm; PIABS	Seed wt*pp1-4*-linked association	3	NR	2.43–4.52	38–41		NR	Candidate locus (single study)	NR	
	Pn; ΦPSII	*qPNC1.1* *qPND2.1*	4; 17	NR	8.01; 6.84	NR	184 Biparental RIL population	NR	Candidate QTL (single study)	NR	Yin et al. (2010)
	Pn	*qPNE.1*	15	NR	6.26	NR		NR	Candidate QTL (single study)	NR	
	Pn; ETo/TRo; ABS/RC; PIABS; qP; ΦPSII	*qPNO.1*	10	NR	6.66	NR		NR	Candidate QTL (single study)	NR	
	qP	*qqpC1.1*	4	NR	18.87	NR		NR	Candidate QTL (single study)	NR	
	Fv′/Fm′; ΦPSII	*qChl-F2* *qChl-F10*	2; 10	NR	11.45; 18.66	55–69	219 accessions	NR	Candidate QTL (single study)	Chr2: *Glyma.02G304700* (*HY2*): ferredoxin oxidoreductase involved in electron transfer and photoreceptor chromophore biosynthesis; Chr10: *Glyma.10G197700* (*NADP-MDH*): NADP-dependent malate dehydrogenase/lactate-malate dehydrogenase	Yang et al. (2022)
	ΦPSII; qP	*qChl-F14* *qChl-F19-1*	14; 19	NR	11.92; 12.05	55–69		3	Candidate locus (single study; detected in multiple environments/years)	Chr14: *GmRCA14*: Rubisco activase; Chr19: *Glyma.19G061200* (*MDAR4*): monodehydroascorbate reductase 4 with chloroplast/cytoplasmic antioxidant and redox roles supporting electron transport and photoprotection;	
	Fv/Fm; NPQ; qP	*qChl-F4-2*	4	NR	12.40	55–69		3	Candidate locus (single study; detected in multiple environments/years)	Chr4: *Glyma.04G182100*: sugar-insensitive 3 (*SIS3*) homolog involved in sugar signaling and integration with light/hormone pathways	
	NPQ; qP	*qChl-F16*	16	NR	20.41	55–69		3	Candidate locus (single study; detected in multiple environments/years)	Chr16: *Glyma.16G010000* (*JAZ6*): jasmonate ZIM-domain protein 6	
Leaf gas exchange	Ci; Cond; Pn; Tr	*GmCDC48*/*Glyma.03G182800*	3	NR	NR	50.28–85.20		2	Candidate locus (single study; multiple traits)	Chr3: *GmCDC48* (*Glyma.03G182800*): cell-cycle protein	Hu et al. (2024)
	Pn; Cond; Ci	*QPH-18-3* (1 SNP)	18	NR	30.15; 8.53	62.90–81.95 (219 accessions); 64.56–74.68 (RIL)	219 accessions/152 F_8:12_RILs (Bogao × NN94-156)	NR	Functionally validated locus	Chr18: *GmFtsH25* (*Glyma.18G065600*): FtsH protease; chloroplast structure	Wang et al. (2023)
	Ci (intercellular CO₂ concentration), low-P/normal-P ratio	*qP13*	13	NR	14.01	50.52	219 accessions (195 landraces and 24 elite varieties)	NR	Functionally validated locus	*HAB2*, *MKK2*, and *GRF5* (signaling/leaf growth)	Yang et al. (2020)
	Co (stomatal conductance), low-P/normal-P ratio	*qP19*	19	NR	16.75	52.76		3	Candidate locus (single study; detected in multiple environments/years)	*RGLG1*, *NAC* proteins, and *P450* (signaling/stress response)	
	Cond; Tr	*qP1*	1	NR	16.89	NR	219 accessions	3	Candidate locus (single study; detected in multiple environments/years)	*PP2C* and *PSAT1* (kinase regulation; amino-acid metabolism)	Wang et al. (2020a, b, c)
	Pn	*qP16*	16	NR	16.30	NR		NR	Candidate locus (single study)	zinc-finger protein and *GGPS1* (light/hormone signaling; isoprenoid synthesis)	
	Pn	NAM P_n_ locus near *Glyma.15G225000*	15	NR	NR	44	SoyNAM: 383 RILs from 32 families	NR	Candidate locus (single study)	Chr15: *Glyma.15G225000*: NADH oxidoreductase-related protein	Lopez et al. (2019)
	WUE	NAM WUE locus near *Glyma.15G182600*	15	NR	NR	60		Multilocation	Candidate region (single study)	Chr15: *Glyma.15G182600*: glucosyltransferase/sucrose-synthase-related protein	
	Vcmax; Vcmax25	Vcmax: ss1235982898, ss1235984400 Vcmax25: ss1235980760, ss1235986347	Vcmax: 13 Vcmax25: 10; 19	Vcmax: 38.94; 41.36 Vcmax25: 44.70; 8.26	NR	Vcmax: NR; Vcmax25: 25.3	SoyNAM: approximately 5,600 F5-derived RILs from 40 families	2	Candidate MTAs (single mapping study)	Vcmax—Chr13 intervals: *Glyma.13G286500* (PsbQ-like 1); *Glyma.13G302100* (*PSB29*); *Glyma.13G308700* (aconitase 3). Vcmax25—Chr10, 43.2–46.7 Mb: *Glyma.10G205500* (PEP carboxylase 4); Chr19, 6.8–13.3 Mb: *rbcS* cluster (Rubisco small subunits)	Montes et al. (2022)
	Jmax; Jmax25	Jmax: ss1235980832, ss1235980869, ss1235983810, ss1235985627 Jmax25: ss1235980832, ss1235980866, ss1235982976, ss1235985627	Jmax: 10; 15; 18 Jmax25: 10; 13; 18	Jmax-10: 44.79, 48.56; 15: 4.50; 18: 56.03 Jmax25-10: 44.79, 48.55; 13: 42.83; 18: 56.03	NR	Jmax: NR; Jmax25: 39.5		2	Candidate MTAs (single mapping study)	Chr10 intervals: *Glyma.10G225400* (*FBPase* candidate; near *E2*), *Glyma.10G243800* (chlorophyll a/b-binding); Chr15: *Glyma.15G050100* and *Glyma.15G052400* (LHCII); Chr18: *Glyma.18G286500* (*PsbP*-like 1); Chr13 Jmax25 interval: candidate NR	
	Asat	SN*E2* peak/*GPA1* candidate region	17	NR	NR	NR	Japanese mini-core GWAS, 74 accessions	NR	Candidate region (single study)	Chr17: *Glyma.17G226700*: *GPA1*; ABA signaling	Shamim et al. (2022)
	An	ss715622638	15	Chr15: 50.41	NR	64	285 MG V accessions	2 years	Candidate locus (single study)	Chr15: *GLYMA_15G266800* (*AGC1.7*; growth); *GLYMA_15G266700* (*cICDH*	Abendroth-McGhee et al. (2026)
	gs	ss715607346, ss715607358, ss715607371 ss715612621	10; 12	Chr10: 44.21–44.39 Chr12: 35.95	NR	69		2 years; one site	Candidate locus (single study; detected in multiple environments/years)	NR	
	WUEi	ss715612250 ss715628694	12; 18	Chr12: 33.07 Chr18: 1.24	NR	54		2	Candidate locus (single study; detected in multiple environments/years)	Chr12: *GLYMA_12G173200* (*GCPE*/*HDS*; chloroplast); *GLYMA_12G172500* (*PIP2B*; water transport)	
	Ci	ss715597729, ss715597730, ss715597732 ss715607358 ss715612250 ss715628694	7; 10; 12; 18	Chr7: 38.18–38.19 Chr10: 44.29 Chr12: 33.07 Chr18: 1.24	NR	49		2	Candidate locus (single study; detected in multiple environments/years)	Chr7: *GLYMA_07G209400* (*OST1*; stomatal movement/water stress); Chr12: *GLYMA_12G173200* (*GCPE*/*HDS*	
Canopy spectral characteristics	PRI	PRI locus 14 PRI locus 15	19; 20	NR	3.70; 3.28	69	373 accessions	NR	Candidate locus (single study)	Chr19: *Glyma19g44010*: *VDE*; NPQ; Chr20: *Glyma20g23930* and *Glyma20g24140*: monooxygenases; carotenoid synthesis	Herritt et al. (2016)
	Extracted, meter, and image-based chlorophyll	Chlorophyll locus cluster, *Gm15* Chlorophyll locus cluster, *Gm19*	15; 19	NR	NR	15–62	332 accessions	NR	Candidate locus (single study)	Mg-chelatase genes (chlorophyll synthesis)	Dhanapal et al. (2016)
	DGCI	high-effect locus high-effect locus locus 33 high-effect locus	5; 7; 15; 20	NR	NR	57–75 (broad); 17–54 (narrow)	200 MG IV accessions	2	Candidate locus (single study)	Chr5 urease; Chr7 ammonium transport; Chr15 nitrate transport; Chr20 purine transport	Kaler et al. (2020)
	BSP (bottom-leaf SPAD value)	*qBSP1e*, *qBSP1b-1*, *qBSP1b-2* *qBSP2d*, *qBSP2f* *qBSP4f* *qBSP9e*, *qBSP9b* *qBSP11e* *qBSP13a* *qBSP18f*	1; 2; 4; 9; 11; 13; 18	Chr1: 3.04–8.72 Chr2: 7.15–9.69 Chr4: 7.23–7.25 Chr9: 5.55–44.16 Chr11: 26.41–26.57 Chr13: 13.79–13.81 Chr18: 6.01–6.08	5.81–10.56	NR	164 F₁₂ ZH RILs (Zhonghuang 24 × Huaxia 3)	2	Candidate locus (single study)	Chr2: *Glyma02g08910*: Chl a/b-binding; light harvesting; *Glyma02g11110*: protein kinase Chl catabolism	Wang et al. (2020c)
	ASP (average SPAD value)	*qASP1f*, *qASP1b-1*, *qASP1b-2* *qASP2e* *qASP3e* *qASP5d-1*, *qASP5d-2* *qASP6f*, *qASP6d* *qASP7a* *qASP9a* *qASP13b-1*, *qASP13b-2* *qASP15a* *qASP16e* *qASP18c*	1; 2; 3; 5; 6; 7; 9; 13; 15; 16; 18	Chr1: 3.06–46.33 Chr2: 6.91–7.03 Chr3: 35.11–35.11 Chr5: 32.92–36.04 Chr6: 5.91–7.25 Chr7: 16.60–16.89 Chr9: 0.365–0.447 Chr13: 40.53–41.87 Chr15: 16.70–17.35 Chr16: 5.62–5.83 Chr18: 55.85–55.90	5.84–16.55	NR		3	Candidate locus (single study; detected in multiple environments/years)	*PAP*/fibrillin; Chl-binding proteins; Chl turnover/synthesis candidates	
	TSP (top-leaf SPAD value)	*qTSP1b* *qTSP2e* *qTSP6b*, *qTSP6d-1*, *qTSP6d-2*, *qTSP6c* *qTSP7e* *qTSP8f-1*, *qTSP8f-2* *qTSP10b*, *qTSP10c* *qTSP11d* *qTSP16e* *qTSP19f*	1; 2; 6; 7; 8; 10; 11; 16; 19	Chr1: 11.59–24.50 Chr2: 7.15–7.23 Chr6: 16.01–48.62 Chr7: 42.36–42.57 Chr8: 2.24–4.82 Chr10: 1.09–5.95 Chr11: 31.43–32.00 Chr16: 5.55–5.61 Chr19: 40.86–40.89	5.61–11.97	NR		3	Candidate locus (single study)	Chr1: *Glyma01g15506*: *PAP*/fibrillin; plastid pigment; Chr2: *Glyma02g08910*: Chl a/b-binding	
	MSP (middle-leaf SPAD value)	*qMSP1c* *qMSP3e* *qMSP6e−1*, *qMSP6e−2* *qMSP7f-1*, *qMSP7f-2*, *qMSP7a-1*, *qMSP7a-2* *qMSP8b* *qMSP14b* *qMSP15a-1*, *qMSP15a-2* *qMSP16d* *qMSP18f-1*, *qMSP18f-2*, *qMSP18d* *qMSP19f*, *qMSP19d*	1; 3; 6; 7; 8; 14; 15; 16; 18; 19	Chr1: 51.90–52.20 Chr3: 34.59–34.70 Chr6: 16.71–43.78 Chr7: 3.90–16.89 Chr8: 2.72–2.74 Chr14: 6.76–7.01 Chr15: 16.70–31.27 Chr16: 1.52–1.54 Chr18: 3.18–47.67 Chr19: 38.71–41.01	5.59–15.50	NR		3	Candidate locus (single study; detected in multiple environments/years)	Chr7: *Glyma07g15960*: N-regulatory protein Chl synthesis; Chr15: *Glyma15g19670*: *ALDH*Chl catabolism; *Glyma15g19810*: Chl a/b-binding	
	Chl_A/B (chlorophyll a/b ratio)	*qChl_A*/B2f *qChl_A*/B7d *qChl_A*/B11e−1, *qChl_A*/B11e−2 *qChl_A*/B12e	2; 7; 11; 12	Chr2: 15.25–15.42 Chr7: 16.60–16.89 Chr11: 2.81–26.43 Chr12: 32.48–33.70	7.16–9.72	NR		3	Candidate locus (single study)	Chr7: *Glyma07g15960*: N-regulatory protein annotated for Chl synthesis	
	Chl_B (chlorophyll b content)	*qChl_B2f* *qChl_B7e−1*, *qChl_B7e−2* *qChl_B10d* *qChl_B15f-1*, *qChl_B15f-2* *qChl_B17f* *qChl_B19e*	2; 7; 10; 15; 17; 19	Chr2: 17.13–26.39 Chr7: 16.60–36.69 Chr10: 4.24–4.44 Chr15: 16.70–49.53 Chr17: 36.49–36.53 Chr19: 31.24–32.80	5.10–12.75	NR		6 (detected at R4, Zengcheng)	Candidate QTL (single study)	Chr7: *Glyma07g15960*: N-regulatory protein Chl synthesis; Chr15: *Glyma15g19670*: *ALDH*Chl catabolism; *Glyma15g19810*: Chl a/b-binding	
	Chl_A (chlorophyll a content)	*qChl_A3d* *qChl_A4e* *qChl_A8d* *qChl_A15f* *qChl_A18e* *qChl_A19e*	3; 4; 8; 15; 18; 19	Chr3: 21.76–21.97 Chr4: 3.72–3.80 Chr8: 12.31–14.09 Chr15: 49.38–49.53 Chr18: 2.01–2.10 Chr19: 2.59–3.57	6.81–13.55	NR		6 (detected at R4, Zengcheng)	Candidate QTL (single study)	NR	
Morphology-related photosynthetic phenotypes	Canopy coverage (CC); speed of canopy closure	35 dynamic CC loci (10 overlapping known flowering-time/plant-height QTLs; 25 novel)	Multiple	Multiple loci	NR	NR	1,303 accessions (903 landraces; 400 improved cultivars)	2	Candidate loci (single study; repeated across developmental time points; 2 loci showed selection evidence)	*E2* (*GIGANTEA*) among previously reported overlapping loci	Li et al. (2023)
	Canopy coverage (CC); average canopy coverage (ACC)	Gm01_50911939_C_T; Gm05_37467797_A_G; Gm06_14104090_T_C; Gm09_4034850_C_T; Gm010_44120764_T_C; Gm010_44630777_C_A; Gm019_1586092_T_C	1; 5; 6; 9; 10; 19	50.91; 37.47; 14.10; 4.03; 44.12–44.63; 1.59	NR (ACC allelic effect: −0.65 to 1.34 percentage points)	77 (ACC)	SoyNAM, 5,555 RILs from 40 families	3	Candidate QTLs (single study; detected across multiple dates/years)	NR	Xavier et al. (2017)
	Leaf length; width; shape; petiole length	important hotspots	4; 14; 19	NR	3.89–23.13 (study-wide hotspot range)	NR	Two RIL populations: ZH n=164; GB n=256	9	Candidate locus (single study; hotspot)	Chr4: *Glyma04g05840*; Chr14: *Glyma14g07140*; Chr19: *Glyma19g37820*; *Glyma19g39340*	Fang et al. (2019)
	Branch/leaf angle; canopy coverage	*qGmBA19-1*/*qGmCC19-1* region	19	Chr19: 1.63	5.40–5.70	NR	399 MG I accessions	9	Candidate locus (single study; hotspot)	Chr19: Candidate region around rs715633239	Virdi et al. (2023)
	Leaflet shape; canopy coverage	*qGmLS4-2*	4	NR	NR	NR		9	Candidate locus (single study; hotspot)	NR	
	SLA	*qSLA3-46* *qSLA15-129*	3; 15	NR	5.39–6.94	NR	137 RILs (Nandou 12 × Jiuyuehuang)	9	Candidate locus (single study; hotspot)	NR	Liu et al. (2019)
	SLW	*qSLW12-137* *qSLW15-140*	12; 15	NR	4.92–6.91	NR		1	Candidate locus (single study; co-localized region)	NR	
	SLA	ss1235980832; ss1235980866; ss1235985627	10; 18	10: 44.79, 48.55; 18: 56.03	NR	36.4	SoyNAM: approximately 5,600 F5-derived RILs from 40 families	2	Candidate MTAs (single mapping study)	Chr10, 43.2–46.7 Mb: *Glyma.10G225400* (*E2*-linked interval); Chr10, 47.1–50.1 Mb: *Glyma.10G249000* (PSI subunit D-2); Chr18, 54.5–57.5 Mb: *Glyma.18G296900* (Rubisco small chain 1 A)	Montes et al. (2022)
	Heterophylly/leaf shape	*qRLS19* *qRLS20*	19; 20	NR	47.2 (maximum); 27.0 (maximum)	NR	Two biparental RIL populations	1	Candidate locus (single study)	Chr19: Candidate genes in *qRLS19* interval; Chr20: Candidate genes in *qRLS20* interval	Chen et al. (2022)
	SLA	ss715581298 ss715591931 ss715593841, ss715594695 ss715597957 ss715604848 ss715612860 ss715621394 ss715625910 ss715632840, ss715630346 ss715635417, ss715635425	2; 5; 6; 7; 9; 12; 15; 17; 18; 19	Chr2: 14.67 Chr5: 40.00 Chr6: 20.28–47.42 Chr7: 40.31 Chr9: 48.05 Chr12: 38.32 Chr15: 25.37 Chr17: 12.65 Chr18: 8.99–31.84 Chr19: 45.15–45.20	NR	67	285 MG V accessions	1	Candidate locus (single study)	Chr5: *GLYMA_05G221300* (*ARF19*/*IAA22*/*ARF11*; auxin/leaf development); *GLYMA_05G222800* (*NDL1*	Abendroth-McGhee et al. (2026)
	LA	ss715582171 ss715586165 ss715590348 ss715608078, ss715608092, ss715608094 ss715609224 ss715620011 ss715623217 ss715635425 ss715636733	2; 3; 5; 10; 11; 14; 15; 19; 20	Chr2: 37.29 Chr3: 40.58 Chr5: 1.95 Chr10: 50.04–50.18 Chr11: 25.02 Chr14: 0.843 Chr15: 9.22 Chr19: 45.20 Chr20: 1.57	NR	79		1	Candidate locus (single study)	Chr5: *GLYMA_05G019900* (*TCP5*; leaf development); Chr19: *GLYMA_19G194500* (*ABI5*	
	LM	ss715590348 ss715593335 ss715604845 ss715624847 ss715632853 ss715635425	5; 6; 9; 16; 18; 19	Chr5: 1.95 Chr6: 15.22 Chr9: 48.00 Chr16: 36.44 Chr18: 9.07 Chr19: 45.20	NR	77		1	Candidate locus (single study)	Chr5: *GLYMA_05G019900* (*TCP5*; leaf development); Chr18: *GLYMA_18G089400* (*FTSZ1-1*	

ABS/RC, absorbed energy flux per reaction center; An, net CO₂ assimilation rate; Asat, light-saturated net photosynthetic rate; Chl, chlorophyll; Chl_A, chlorophyll a content; Chl_A/B, chlorophyll a/b ratio; Chl_B, chlorophyll b content; Ci, intercellular CO_2_ concentration; Co/Cond/gs, stomatal conductance; DGCI, dark green color index; DI_0_/RC, dissipated energy flux per reaction center; ET_0_/ABS, electron-transport yield per absorbed photon; ET_0_/RC, electron-transport flux per reaction center; ET_0_/TR_0_, electron-transport efficiency per trapped exciton; Fᵢ and Fⱼ, fluorescence intensities at the I and J steps, respectively; Jmax, maximum electron-transport rate; LA, leaf area; LM, leaf mass; LOD, logarithm of odds; M_0_, initial slope of fluorescence transient; MSP, middle-leaf SPAD value; NAM, nested association mapping; NPQ, non-photochemical quenching; NR, not reported; PIABS, performance index on an absorption basis; PRI, photochemical reflectance index; PSII, photosystem II; PVE, phenotypic variance explained; qP, photochemical quenching; QTL, quantitative trait locus; R^2^, coefficient of determination; RC, reaction center; RIL, recombinant inbred line; SLA, specific leaf area; SLW, specific leaf weight; SPAD, Soil Plant Analysis Development chlorophyll-meter index; TR_0_/RC, trapped energy flux per reaction center; Tr, transpiration rate; Vcmax, maximum Rubisco carboxylation rate; Vⱼ, relative variable fluorescence at the J step; WUE, water-use efficiency; WUEi, intrinsic water-use efficiency; φD_0_, quantum yield of energy dissipation; φE_0_, quantum yield of electron transport; φP_0_, maximum quantum yield of primary photochemistry; ψ_0_, probability that a trapped exciton transfers an electron beyond Qₐ

In the SoyNAM study (Montes et al. 2022), several significant marker-trait associations (MTAs) were found to be clustered on chromosomes 10 (LG O), 13 (LG F), and 18 (LG G). Notably, some of these MTAs co-located with loci previously associated with yield-related traits, including seed yield, plant height, lodging, and canopy coverage (Diers et al. 2018; Xavier and Rainey 2020). While this co-localization does not imply causality, it suggests that selection for yield-related traits may have indirectly captured alleles influencing PE through genetic linkage or pleiotropy. However, such indirect selection is inherently imprecise and context-dependent, as correlated responses may vary across environments, growth stages, and genetic backgrounds. These findings, therefore, highlight the opportunity and need for direct integration of high-resolution phenotyping and genomics to explicitly target photosynthetic traits. It enables more precise and effective improvement of PE beyond what can be achieved through indirect selection only.

The major candidate gene *Glyma.10g225400* encoding fructose−1,6-bisphosphatase (*FBPase*) was associated with J_max_, consistent with its central role in RuBP regeneration. This gene is of particular interest because *FBPase* plays a key role in RuBP regeneration within Calvin-Benson cycle. Experimental studies have shown that elevating *FBPase* activity can enhance carbon flux through cycle, leading to improved CO_2_ assimilation, PE and biomass accumulation (Croce et al. 2024; Tamoi et al. 2006). In the same chromosome 10, the study also highlighted E2 (*Glyma.10g221500*)- a GIGANTEA homolog that is well known for controlling flowering time and maturity group adaptation in soybean. However, given E2’s primary role in phenology, the observed signal can be more plausibly interpreted as an indirect effect of maturity and canopy duration on PE and yield, and/or linkage to nearby causal gene(s) affecting leaf C and N traits. In this context, loci in the E2 region could influence PE and seed yield indirectly by modifying leaf and canopy attributes, such as chlorophyll content, leaf N concentration and canopy duration, that regulate PE and seasonal carbon gain.

Despite these insights, most reported associations exhibit small effect sizes (R^2^) and are highly population- and study-specific, consistent with previous findings showing that QTLs for photosynthetic traits in soybean are generally modest, unstable, and difficult to reproduce. To date, no major or consistent gene controlling PE has been consistently identified across genetic backgrounds and environments. These observations highlight that improving PE will require breeding approaches that move beyond single-locus targets, and instead integrating multi-omics with accurate, high-throughput phenotyping of biologically meaningful secondary traits to better resolve complex genetic architecture underlying PE.

Wang et al. (2020a) identified two novel and relatively stable genomic regions *qp1* and *qP16* associated with net photosynthesis rate (Pn) and g_s_. The *qP1* region harbored candidate genes, including *Glyma.01g158000* (protein phosphatase 2 C) and *Glyma.01g159800* (*Phosphoserine aminotransferase*), while *qP16* overlapped with previously reported QTLs for plant height, pod shattering, and seed weight. Two key candidates in this regions, *Glyma.16g141100* (zinc-finger protein family associated with soybean pods) and *Glyma.16g141700* (*a geranylgeranyl pyrophosphate synthase*) were proposed to underlie these associations.

Among the numerous QTLs reported for photosynthesis-related traits in soybean, *GmFtsH25*, underlying the environmentally stable QTL *QPH-18-3*, remains the only gene that has been cloned and functionally characterized to date (Wang et al. 2023). This gene has recently been associated with g_s_, net photosynthetic rate (Pn), and intercellular CO_2_ concentration (Ci) via GWAS and linkage mapping approaches. Soybean lines overexpressing *GmFtsH25* exhibited enhanced growth, higher photosynthetic rates, and improved yield components under controlled environment (Wang et al. 2023). Nevertheless, large-scale, multilocation field validation across diverse genetic backgrounds is still needed to confirm its contribution to agronomic yield improvement under realistic production environments.

Table 1 summarizes these loci, but their poor cross-environment reproducibility raises a critical question: how can robust photosynthetic QTLs be mined when G×E interactions erode effect sizes and reverse allelic rankings across environments? Photosynthetic QTLs in soybean are characterized by small effect sizes and poor cross-environmental stability. Hu et al. (2024) reported heritability estimates for chlorophyll fluorescence parameters ranging from 16.8 to 9038% across environments and growth stages. Gas exchange traits showed moderate to high heritability at R2 (50.3–85.2%), but these values decline sharply by R6 (15.6–34.6%). Hao et al. (2012) similarly documented low heritability (37.6–47.3%) for fluorescence parameters across 168 landraces, attributing the residual variation largely to G×E effects. In SoyNAM population, MTAs for V_max_ and J_max_ co-localized with the E2 maturity locus, indicating that photosynthetic signals may partly reflect indirect phenological variation rather than intrinsic carbon fixation capacity (Montes et al. 2022). Collectively, these findings demonstrate that G×E interactions substantially erode the genetic signal for photosynthetic traits.

G×E interactions in soybean frequently produce crossover effects that reverse allelic rankings between environments. Happ et al. (2021) evaluated 213 lines across 11 environments and found that 73.21% of G×E QTLs caused crossover interactions. Marker×year×location effects were especially prone to rank changes. For photosynthetic traits, this instability is compounded by developmental shifts in source-sink relationships. Trait expression at early reproductive stages often fails to predict late-season performance due to cumulative temperature and water stress (Hu et al. 2024). Consequently, QTLs with strong effects in one environment may become negligible or change direction under different field conditions.

Repeated phenotyping across contrasting environments is the first step toward robust locus discovery. Multi-environment trials separate stable QTLs from environment-specific noise. The SoyNAM population was phenotyped under both irrigated and rainfed conditions, enabling identification of photosynthetic loci that maintained significance across water regimes (Montes et al. 2022). Yang et al. (2020) screened 219 accessions for photosynthetic traits under low and high phosphorus levels across multiple environments. This design revealed P-responsive QTLs that single-environment analysis would have missed. For soybean, photosynthetic traits should be evaluated across at least three environments differing in moisture, temperature, or nutrient status. Such replication captures stable genetic variation and reduces false positives from single-environment snapshots. Joint analysis across multiple populations and environments increases the power to detect stable photosynthetic loci. Single panel GWAS is constrained by restricted allelic diversity and population structure. Multi-parent population such as SoyNAM combine 40 biparental families and capture broader genetic variation than single crosses (Montes et al. 2022). When summary statistics from multiple environments are analyzed jointly, loci with small but consistent effects across populations become detectable. This approach is particularly relevant for photosynthetic traits where individual environment effect sizes often fall below significance thresholds.

Time-series phenotyping captures the developmental dynamics of photosynthetic QTLs that single time-point measurements miss. In wheat, daily phenotypic profiling of a MAGIC population revealed that QTLs for plant height and senescence changed in significance across the growing season, with some loci becoming active only after 136 days (Camargo et al. 2018). Functional mapping of longitudinal data can model the onset, peak, and decay of QTL effects across developmental stages. For soybean, applying such dynamic mapping to repeated canopy reflectance or fluorescence measurements during R2–R6 could separate transient environmental responses from genetically controlled photosynthetic trajectories. Reaction-norm models that incorporate marker-by-environment covariance functions capture QTLs that are invisible to conventional single-environment GWAS (Jarquín et al. 2014). In soybean, mixed models with marker-by-environment interactions have identified crossover QTLs that are not captured by traditional stability estimators (Happ et al. 2021). For photosynthetic traits, replacing single-environment analysis with multi-environment mixed models or reaction-norm frameworks is necessary to recover robust loci. Together, these useful strategies including multi-environment phenotyping, joint population analysis, dynamic time-series mapping, and explicit G×E modeling, shift photosynthetic genetics from descriptive QTL cataloging to robust locus discovery. Only through such integrated approaches can the small, unstable effects underlying soybean photosynthetic efficiency be translated into breeding-relevant targets.

Photosynthesis is a dynamic and environmentally sensitive biological phenomenon, and its genetic expression is strongly influenced by fluctuating field conditions. While the identification of candidate loci and genes has advanced our understanding of the genetic basis of photosynthetic traits, their effective deployment in breeding depends on validation through robust field studies, repeated multi-environmental phenotyping, and explicit modeling of G×E interactions. These strategies are effective to overcome the small effect sizes and poor cross-environment stability of photosynthetic QTLs. Overall, these advances demonstrate substantial natural variation and a highly complex genetic architecture underlying PS-related traits in soybean, including variations in V_cmax_, J_max_, P_n_, and Fv/Fm. Despite this progress, a critical gap remains in linking this genetic variation to seed yield under realistic field conditions. Addressing this gap is essential not only for translating physiological and genetic insights into breeding strategies, but also for sustaining long-term genetic gains in soybean, where continued improvement is increasingly constrained by a narrow genetic base and diminishing returns from conventional selection.

## Photosynthesis-seed yield relationships in soybean: evidence, stage dependency, and practical implications

As previously discussed, crop yield potential (Y), in general, can be partitioned into three physiological components: light interception efficiency, its conversion efficiency (radiation use efficiency), and harvest index. This scenario serves as foundational understanding of the biological determinants of yield across crop species, including soybean. However, in practical agronomic and breeding contexts, seed yield is the result of complex interactions among genotype (G), dynamic environmental conditions (E), and diverse management practices (M) throughout the growing season (Chiozzaet al. 2024; Croce et al. 2024). These interactions modulate each of the yield components (such as seed size, weight, and number per unit area), often making it difficult to directly attribute yield variation to individual physiological traits. Therefore, understanding how photosynthetic traits relate to seed yield within this G**×**E**×**M framework is critical for effectively integrating into breeding strategies aimed at improving soybean productivity (Hajjarpoor et al. 2022). The environmental conditions include soil characteristics (composition, texture, porosity, pH, moisture, nutrients), climatic conditions (temperature, humidity, precipitation, solar radiation, photoperiod, wind), and other biological factors (pathogens, microbiome, predators, insects-pests). Genetically, seed yield is a highly complex quantitative trait controlled by numerous genes/QTLs, each with small to moderate effects, whose expression and contribution to yield are further influenced by environmental variation, and genotype by environment interactions (G**×**E) (Diers et al. 2018; Kabelka et al. 2004; Chung et al. 2003; Guzman et al. 2007; Wang et al. 2014; Xavier and Rainey 2020; Fang et al. 2017; Han et al. 2012, 2024; Hu et al. 2014; Fox et al. 2015; Li et al. 2024; Xavier et al. 2018).

Seed yield in soybean is primarily determined by its components, including seed number (SN), seed weight (SW), and pod number per plant (PN), with studies suggesting that pod number, i.e., more pods per unit area, may have particularly stronger influence on yield (Schou et al. 1978; Egli 2013; Kahlonet al. 2011). Each of these components is influenced by varying degrees via physiological parameters such as ***ε***_***i***_, RUE, and ***ε***_***p***_. Understanding how these yield components respond to PE under different environmental conditions is critical for bridging physiological efficiency with genetic gain (yield improvement over time). Light interception efficiency (***ε***_***i***_) is strongly affected by canopy structure, leaf area index, and plant architecture. Greater light interception increases assimilate availability during flowering and pod setting, thereby enhancing pod and seed numbers per unit area. Therefore, traits such as node number, branching, and plant height can contribute to higher ***ε***_***i***_ (Feng et al. 2019; Ashley and Boerma 1989; Theeuwen et al. 2022). Radiation use efficiency (RUE) or E_c_, on the other hand, determines how efficiently the intercepted light is converted into biomass (both vegetative and reproductive tissues). It is strongly linked with PE, stomatal conductance, chlorophyll content, and source-sink dynamics (Table 2). RUE affects both seed weight and seed number, depending on source availability during seed filling (Burgess and Degen 2023; Cho et al. 2023; Montes et al. 2022). There is substantial scope to improve RUE in C3 crops, including soybean and wheat (Croce et al. 2024; Faralli and Lawson 2020). Evidence from wheat supports this potential, where long-term genetic gains were largely attributed to an increase in RUE. Also, genetic variation for this trait showed high heritability and strong correlation (Li et al. 2022). Net photosynthesis and biomass accumulation were key drivers of RUE, and physiological traits (leaf water content, morphology, and chlorophyll content) represent useful targets for genomics-based selection strategies, including genomic prediction, to accelerate improvement of PE. These findings highlight that breeding progress for RUE is achievable when such traits are incorporated into integrative genome prediction models, and that improving source capacity through RUE optimization has the potential to contribute meaningfully to future yield gains.

**Table 2:** Summary of major studies in soybean investigating photosynthesis-related traits and proxies in relation to seed yield components

Plant Material	Traits	Growth stage	Screening environment(s)	Yield correlation (r)		References
				Phenotypic	Genotypic	
SoyNAM subset (5171 F5-derived RILs plus 40 founder lines)	Vcmax25; Jmax25;Chl; C%; N%; SLA	R4–R5 (seed filling)	2	Chl + 0.255; N% + 0.20; Vcmax25 + 0.06; Jmax25 − 0.04	Chl + 0.45; C% + 0.66; N% + 0.83; SLA + 0.72; Jmax25 − 0.62; Vcmax25 − 0.09 (NS	Montes et al. 2022
Diverse association panel (1303 accessions)	Time-series canopy coverage (CC; canopy-closure rate)	17 time points during canopy development	1 (Nanchang, China; field; 2020; UAS phenotyping	CC significantly correlated with GYPP (r = 0.40–0.55); top 15% yielders had higher max CC	NR	Li et al. 2022
SoyNAM (5555 F5-derived RILs; 40 families)	Average canopy coverage (ACC)	V2–R5	3	Pearson + 0.63; Spearman + 0.70	+0.88	Xavier et al. 2017
SoyNAM subset (383 RILs from 32 families)	Leaf photosynthetic rate (A; intrinsic WUE)	Late R4–early R5	3	A − 0.02; WUE NR	A +0.80; WUE NR	Lopez et al. 2019
16 experimental soybean lines	Apparent photosynthesis; SPAD; specific leaf weight	R2, R4 and R5	4	Apparent photosynthesis + 0.54	NR	Thompson et al. 1995
12 varieties	Photosynthetic rate per unit leaf area (PA; leaf N)	R4–R5	3	Leaf N + 0.75; PA + 0.78	NR	Buttery et al. 1981
Diverse association panel (219 accessions)	ABS/RC; Chl; ET_0_/ABS; ET_0_/TR_0_; Fv/Fm; PIABS; Ci; Cond; Pn; Tr	R2 and R6	4 field environments for seed yield; 3 net-room experiments for photosynthetic traits	+ 0.10 to + 0.36 (trait-dependent)	NR	Hu et al. 2024
Four soybean varieties	Leaf chlorophyll content	R6	1	+0.893	NR	Gureeva and Levakova 2023
One variety (Tiefeng 40 under biochar × fertilizer treatments)	SPAD; Pn; Cond; Ci; Tr; LDW; Chl; LAI; Fv/Fm; ABS/RC; PIABS; DFTotal	R2	1 long-term field experiment	SPAD + 0.83; Pn + 0.78; Cond + 0.70; Ci − 0.70; Tr + 0.75; LDW + 0.88; Chl + 0.82; LAI + 0.85; Fv/Fm + 0.76; ABS/RC − 0.75; PIABS + 0.80; DFTotal + 0.78	NR	Wu et al. 2024
Seven varieties	SPAD; leaf area index (LAI)	SPAD: R6; LAI: R3, R5 and R7	2	SPAD at R6 + 0.854; LAI at R3 + 0.776, R5 + 0.782 and R7 + 0.779	NR	Sun et al. 2025
F4-selected lines and their derived F6 progenies from one soybean cross	Canopy-apparent photosynthesis (CAP)	R4-R5 (seed development	2	+ 0.28 to + 0.66	NR	Ashley and Boerma 1989
38 varieties released from 1923–2005	Pn; SLW; Chl; Gs; Ci; Ci/Ca; Pn/Ci; Tr; WUE	R2 and R4	2	Pn + 0.610**; SLW + 0.446**; Chl + 0.308; Gs + 0.161; Ci − 0.282; Ci/Ca − 0.349*; Pn/Ci + 0.521**; Tr + 0.628**; WUE − 0.517**	NR	Liu et al. 2012
24 varieties released from 1923–2007	Seasonal light interception efficiency; radiation use efficiency; harvest index	Season-long measurements	2	2012: light interception + 0.42*; RUE + 0.70***; HI + 0.86***. 2013: light interception + 0.56**; RUE + 0.29 (NS; HI + 0.79***	NR	Koester et al. 2014
97 soybean genotypes	UAV-derived canopy coverage	V3–V4, V5–V6, V7–V8 and V9–R1	2	− 0.36, + 0.41, + 0.49 and + 0.46, respectively, in one environment	+ 0.76 at flowering in one environment	Casagrande et al. 2022

A, leaf photosynthetic rate; ABS/RC, absorption flux per reaction center; ACC, average canopy coverage; C, carbon concentration; CAP, canopy-apparent photosynthesis; CC, canopy coverage; Chl, chlorophyll content; Ci, intercellular CO₂ concentration; Cond/Gs, stomatal conductance; DF Total, total driving force; ET₀/ABS, quantum yield for electron transport; ET₀/TR₀, probability that a trapped exciton moves an electron beyond Qₐ; Fv/Fm, maximum quantum yield of PSII; GYPP, grain yield per plant; Jmax25, maximum electron-transport rate at 25 °C; LAI, leaf area index; LDW, leaf dry weight; N, nitrogen concentration; NR, not reported; NS, not significant; PA, photosynthetic rate per unit leaf area; PIABS, performance index on an absorption basis; Pn, net photosynthetic rate; RIL, recombinant inbred line; SLA, specific leaf area; SLW, specific leaf weight; SPAD, chlorophyll-meter reading; Tr, transpiration rate; UAS/UAV, unoccupied aircraft system/unoccupied aerial vehicle; Vcmax25, maximum Rubisco carboxylation rate at 25 °C; WUE, water-use efficiency. *P < 0.05; **P < 0.01; ***P < 0.001

Optimizing canopy architecture is a critical strategy to improve PE and seed yield in soybean (Long et al. 2006). Architectural traits such as leaf morphology, petiole length, internode length, and branching significantly influence light distribution within the canopy, thereby impacting Ei and RUE (Liu et al. 2017; Sreekanta et al. 2024). For example, narrower leaves, shorter petioles, and more upright leaves promote deeper light penetration, which can enhance photosynthetic activity in lower canopy parts (such as pods and seeds), thereby contributing to yield formation (Soares et al. 2021; Cho et al. 2023). Recent historical analyses also showed that yield gains in Northeast China over the past 80 years were accompanied by improvements in canopy RUE, particularly during reproductive stages. These improvements were driven by optimized canopy architecture (reduced branching, higher chlorophyll) and enhanced biomass accumulation post-reproductive growth stage R3 (Waqar et al. 2025).

While all three components (Ei, RUE, and HI) are positively associated with yield, empirical and genetic studies indicate only moderate correlations among them, reflecting distinct underlying biological processes and environmental sensitivities (Lopez et al. 2021; 2022). Ei is primarily governed by canopy architecture and leaf area dynamics, RUE by PE and stress tolerance, and HI by sink strength, pod set, and assimilate partitioning. This partial independence suggests that simultaneous improvement of Ei, RUE, and HI remains physiologically feasible and offers multiple, complementary avenues for yield improvement.

In modern soybean cultivars, Ei during the key reproductive stages often exceeds 90% of incident PAR, whereas RUE typically ranges from ~ 0.8–1.5 g biomass MJ⁻^1^ of intercepted radiation, depending upon genotype and environment (Xu et al. 2021; Lopez et al. 2021). Harvest index in contemporary production systems commonly ranges between 0.35 and 0.45, values that remain well below proposed theoretical upper limits for legumes (Jiang et al. 2019; Löwik 2023; Naydenova et al. 2022). Higher HI indicates more biomass goes into seeds, which can impact seed weight and number. Together, these benchmarks indicate that historical yield gains have resulted from modest, cumulative improvements across Ei, RUE, and Hi rather than optimization of any single component, and the scope of further improvement remains, particularly under stress-prone environments.

Unlike cereals, where semi-dwarfism directly improved HI by reducing lodging and enabling high planting densities, soybean plant height and HI are closely linked to growth habit and phenological plasticity. The widespread adoption of indeterminate cultivars reflects greater yield stability and capacity for compensation under variable environments. Consequently, improving HI in soybean is more likely to succeed through architectural ideotypes that maintain indeterminate growth while incorporating compact canopy traits such as reduced internode length, improved lodging resistance, optimal branching, and prolonged green leaf area during seed filling, rather than through a shift toward determinacy. Such traits influence Ei and RUE while safeguarding assimilates availability to reproductive sinks, providing a physiologically coherent route to incremental yet durable improvements in HI and overall yield potential.

Previous research has shown a positive relationship between PE and seed yield in soybean across multiple studies. However, it is important to note that these studies differ widely in their target photosynthetic trait(s), measurement growth stages (within reproductive stage), screening environments, and yield components considered (Table 2). Most yield-correlation studies were focused on leaf chlorophyll (Chl) traits such as SPAD, Chl a, Chl b, and Chl a/b (Montes et al. 2022; Lopez et al. 2019; Wu et al. 2024; Liu et al. 2012; Hengshan et al. 2001). The Chl traits have consistently shown moderate to high correlations with soybean seed yield (r = 0.26–0.90), suggesting a positive relationship across diverse environmental conditions. Over 82 years of genetic improvement in China, soybean varieties exhibited increased net photosynthetic rate (P_n_), g_s_, chlorophyll content, and specific leaf weight (SLW), which correlated positively with higher seed yields (Liu et al. 2012). Notably, yield improvement was also accompanied by a reduction in days to maturity. Similarly, genotypes with high SLW demonstrated greater P_n_ and comparable yields in adapted environments (Thompson et al. 1995). Photosynthesis at individual nodes has been shown to drive rapid increase in seed number with increasing carbon input, up to a saturation threshold (Bruening and Egli 1999). More recent studies confirm that photosynthetic traits, including LAI, chlorophyll content, g_s_, and P_n_, have improved through decades of breeding (Bao et al. 2021). Moreover, newer varieties exhibit better responsiveness and stability under varying fertilizer regimes, maintaining PE under both optimal and high nutrient conditions, likely due to enhanced root traits and nutrient-use efficiency (Bao et al. 2021).

Building on this, Hu et al. (2024) reported positively correlations between soybean yield components and several chlorophyll fluorescence parameters (r = 0.13–0.36) across multiple environments. Specifically, SW was positively correlated with Chl, Fv/Fm, ET0/TR0, ET0/ABS, PIABS and P_n_, while showing negative correlations with ABS/RC and intercellular CO_2_ concentration (Ci). Seed yield per plant (SPP) showed a similar pattern of positive correlations with multiple physiological parameters, but a negative relationship with ABS/RC. Earlier field studies also reported significant associations between seed yield and photosynthetic traits in soybean (Thompson et al. 1995; Ma et al. 1995; Buttery et al. 1981; Liu et al. 2005). However, these studies often involve a limited number of genotypes, which may constrain their statistical power and the broader inferences. Notably, some studies have found weak or absent phenotypic correlations between PE and seed yield. For instance, Lopez et al. (2019) observed no phenotypic association between net assimilation rate (P_n_) and seed yield, despite a strong genetic correlation (r = + 0.80), suggesting that non-genetic factors substantially influence P_n_. Similarly, Montes et al. (2022) evaluated key photosynthetic traits in a large and genetically diverse soybean panel and found that the rate limiting steps of photosynthesis such as V_cmax25_ and J_max25_ showed low or even negative correlations with seed yield under field conditions. In particular, J_max25_ showed a strong negative genetic correlation with yield (r = − 0.62), while Vmax25 showed only an indirect relationship through its positive correlation with J_max25_ (r = 0.36). Among phenotypic correlations, chlorophyll content displayed the highest, though still moderate correlation with yield (r = 0.26). In contrast, other traits such as chlorophyll content (r = 0.45), leaf N (r = 0.83), leaf C (r = 0.66) and specific leaf area (r = 0.72) showed stronger genetic correlation with seed yield, indicating their potential utility as indirect selection targets for physiological breeding. Interestingly, these observations contrast with earlier findings or modeling simulation frameworks, where increasing V_cmax_ was projected to enhance yield (Long et al. 2006; Ort et al. 2015). Such differences may arise due to substantial variations in growing environments, source-sink dynamics, measurement approaches or complex genetic bases of photosynthesis and seed yield under field conditions. Therefore, while V_cmax_ and J_max_ remain important from a mechanistic perspective, their value as direct selection traits for yield improvement in soybean may be context-dependent. Further validation across environments and breeding populations is warranted to better understand their role within agronomic frameworks.

Beyond leaf photosynthesis, soybean pods and seeds also contribute meaningfully to canopy photosynthesis, and final seed yield (Burgess and Degen 2023). Although most research has focused on photosynthesis in leaf tissues, studies show that pod and seed tissues, which contain lower concentrations of Rubisco than leaves, also perform photosynthesis and can assimilate CO_2_. Light exclusion soybean experiments by Cho et al. (2023) revealed a 13–14% reduction in seed weight when pods were shaded, underscoring their contribution to final yield formation. While shading reduced chlorophyll content and PE in pods, non-foliar photosynthesis was found to offset up to 81% of respiratory carbon losses and contributed approximately 9% of total canopy photosynthesis. These findings emphasize the potential of optimizing light distribution and RUE, particularly in the lower canopy, as a strategy to improve seed yield.

Canopy photosynthesis represents the combined photosynthetic activity of all photosynthetic tissues, which drives the production of vegetative biomass and seed yield in crops. Throughout the growing season, canopy photosynthesis adjusts in response to changing environmental conditions and rarely reaches saturation, even under full sunlight, due to shading effects from neighboring plants (Zhu et al. 2012). Canopy-apparent photosynthesis (CAP) has been shown to correlate positively with seed yield (r = 0.28 to 0.66) during early seed development stage (R4–R5) in soybean (Wells et al. 1982; Harrison et al. 1981; Ashley and Boerma 1989). These studies provide empirical evidence linking canopy-level RUE to seed yield formation. Additionally, photosynthetically active radiation (PAR) has also been reported to influence yield components in soybean such as seed number and pod number (Feng et al. 2019; Liu et al. 2017), suggesting relatively higher light intensities (400–500 umoles/m^2^/sec) can enhance these yield components via more biomass accumulation. Similarly, a study by Kantolic et al. (2013) found that extended photoperiod during R3–R6 stage, increased pod and seed numbers per unit area in soybean, further reinforcing importance of intercepted radiation in shaping soybean yield potential.

Collectively, these findings underscore the complex yet exploitable relationships between photosynthetic traits and seed yield in soybean. Traits such as SPAD, chlorophyll content, specific leaf weight, PSII efficiency, net assimilation rate (P_n_) show moderate to strong associations with seed yield, whereas biochemical parameters like V_cmax25_ and J_max25_ seem less reliable predictors under field conditions, thereby warranting further investigation. The combined influence of canopy structure, light distribution and non-foliar tissue photosynthesis highlights the need for a system-level approach to improve RUE and seed yield. Together, these insights provide the physiological foundation for breeding strategies aimed at enhancing soybean productivity through improved PE, especially when combined with high-throughput phenotyping, genomic prediction and G**×**E modeling discussed in the following section.

## Integrating photosynthetic traits into soybean breeding pipelines: from germplasm discovery to genomic-phenomic prediction

Substantial progress has been made in improving different components of soybean yield formation; however, precision breeding for photosynthesis-driven yield enhancement remains a largely untapped area. This can be primarily attributed to the dynamic and complex nature of this fundamental physiological phenomenon, as it involves a multitude of major and minor genes, their interaction with the environment and management practics (G**×**E×M), as well as biochemical pathways (Theeuwen et al. 2022). In C3 plant canopies, the photosynthetic machinery becomes fully saturated at approximately 25% of the maximum available solar radiation, which is a key factor limiting the productivity of these crop plants (Jansson et al. 2018). Understanding the intricate relationship between photosynthetic traits and seed yield components at different growth stages is crucial for utilizing them using modern breeding tools for developing soybean varieties with higher yield potential. Photosynthesis is a multifaceted process, influenced by various factors such as resource use efficiencies for light, water, and nutrients, temperature responses, leaf architecture, and stress tolerance. These factors are independent and vary during crop cycles in magnitude and availability (e.g., light availability) (Flood et al. 2011; Faralli and Lawson 2020). Canopy photosynthesis (CP) is controlled by both microclimate and the photosynthetic properties of individual leaves. While CP can be assessed with specialized chambers (Song et al. 2016), accurate models are needed to dissect key regulatory factors, with existing models focusing on canopy structure and microclimate heterogeneity (Song et al.^2017^; Liu et al. 2021). Recent research on soybean has highlighted the importance of optimal LAI and leaf angle to enhance CP (Tamang et al. 2026). NPQ may offer potential for breeding soybean with improved PE, though variation in NPQ relaxation appeared to be limited in this crop, with the difference in PE majorly linked to Rubisco activation (Wang et al. 2020b). Independent studies have predicted that reducing chlorophyll content could increase nitrogen use efficiency (Sakowska et al. 2018) and simulations in soybean suggest that a 50% reduction in leaf chlorophyll content could result in up to a 9% reduction in leaf nitrogen (Walker et al. 2018).

Transgenic approaches such as the introduction of cyanobacterial enzymes (*SBPase*/*FBPase*) have shown promise to improve CO_2_ assimilation and growth in soybean, particularly under elevated CO_2_ conditions (Köhler et al. 2017). These findings suggest that enhancing RuBP regeneration could help mitigate climate-induced yield losses and emphasize the need for these approaches to be tested under future climatic scenarios (Raines 2022). Also, recent review suggests that increasing the abundance or efficiency of Rubisco itself could provide a more direct route to boosting CP, with modeling indicating ~ 9% CP gain in soybean without additional nitrogen costs (Salesse-Smith et al. 2025). Other transgenic efforts have focused on improving soybean PE by targeting leaf chlorophyll content and accelerating the relaxation of NPQ, with the goal of enhancing seed yield and/or biomass (Croce et al. 2024). For instance, reducing leaf chlorophyll content has been proposed to improve light distribution in monocrop fields, boosting overall PE and seed yield (Blankenship and Chen 2013; Cutolo et al. 2023). Though it showed some success in tobacco and rice, it did not show promise in soybean chlorophyll-deficient mutants, which showed reduced biomass and seed yield (Slattery et al. 2017; Sakowska et al. 2018). It underlies the complex nature of these traits, influenced by factors like gene pathways, chlorophyll level, canopy structure, and environmental conditions (Cutolo et al. 2023). De Souza et al. (2022) reported that overexpressed violaxanthin de-epoxidase (*VDE*), PSII subunit S (*PsbS*) and ZEP (*VPZ* genes) improved biomass and seed yield up to 33% (with an average of ~ 25%) in ‘Maverick’ genotype. However, this study faced substantial criticism due to the lack of agronomically sound experimental design, hypothesis, seed yield measurements, and data interpretation (Sinclair et al. 2023). This underscores the need for large-scale, multi-location field trials to evaluate the impact of such genetic manipulations on agronomically important complex traits like seed yield (Khaipho-Burch et al. 2023a). To ensure robust evaluations of mutants aimed at improving yield, collaboration among plant breeders, agronomists, and crop physiologists is essential for selecting appropriate yield-testing protocols, including plot designs that minimize edge effects, defining optimal plant densities, and standardizing yield parameters (Khaipho-Burch et al. 2023b; Hajjarpoor et al. 2022; Chiozza et al. 2024). These well-designed field trials would also offer an opportunity to test other potential benefits associated with the pale green phenotype, such as improved nitrogen use efficiency.

To effectively screen for functional variation in soybean photosynthesis under dynamic conditions, it is crucial to replicate field-like conditions within controlled environments (Murchie et al. 2018; Cruz and Avenson 2021) for initial screening. This allows high-throughput phenotyping of photosynthetic traits to accurately identify genetic variation suitable for soybean breeding (van Bezouw et al. 2019; Song and Zhu 2024). While progress has been made in measuring several photosynthetic parameters, there are still significant gaps in the ability to efficiently phenotype all relevant traits (Siebers et al. 2021). The development of HTP techniques, especially portable devices and UAVs-based systems, is therefore essential to ensure reproducibility for genetic studies (Cruz et al. 2016).

Soybean offers considerable untapped potential to improve PE and RUE. At the canopy scale, suboptimal light distribution caused by self-shading and canopy architecture suggests scope for modest but meaningful gains in canopy photosynthesis by roughly 5–10% (Sreekantaet al. 2024). At the leaf level, limitations related to electron transport, Rubisco activation, and nitrogen allocation further constrain PE (Lopez et al. 2021). Moreover, a substantial proportion of RUE loss in production environments arises from stress induced declines in photosynthesis and premature senescence, indicating that improving stress-resilient photosynthesis and maintaining green leaf area during seed filling could translate into measurable seed yield gains (Anda et al. 2021; Phillips 2021). Together, these observations highlight multiple, complementary avenues to improve soybean RUE by 15–20%, through which photosynthesis-guided breeding strategies could contribute to enhanced soybean yield potential.

**Fig. 3:**
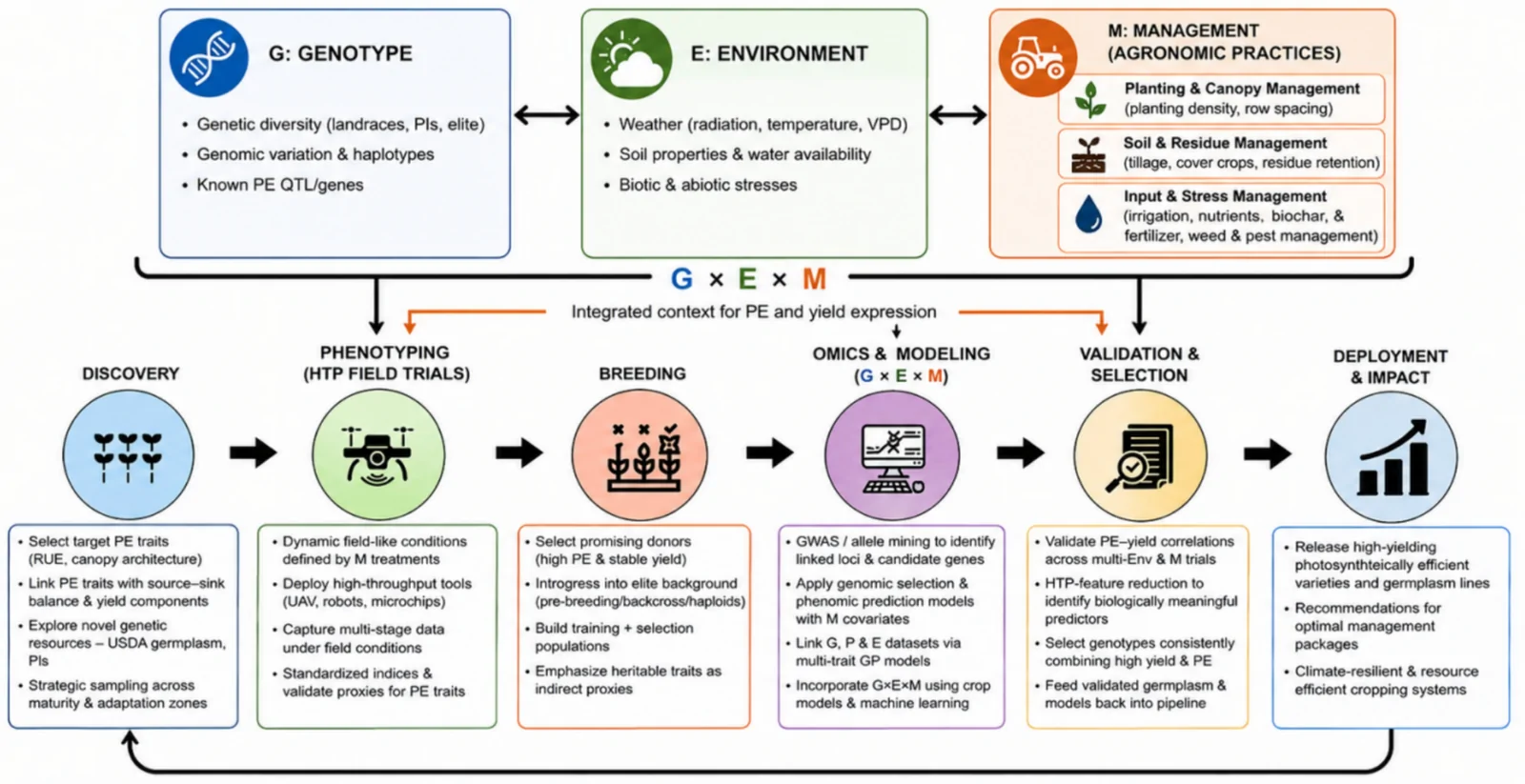
An enhanced strategic pipeline for incorporating photosynthetic traits into soybean breeding under a G×E×M framework. The framework integrates genetic (G), environmental (E), and management (M) factors across discovery, high-throughput phenotyping, breeding, omics- and modeling-based prediction, validation and selection, and deployment. The integrated G × E × M context supports PE trait discovery, field phenotyping, donor selection, genomic and phenomic prediction, validation of PE-yield relationships, and deployment of improved germplasm and management recommendations. PE, photosynthetic efficiency; RUE, radiation use efficiency; PI, plant introduction; HTP, high-throughput phenotyping; GWAS, genome-wide association study; QTL, quantitative trait locus

Despite substantial progress in dissecting the physiological determinants of PE, the genetic architecture of photosynthetic traits (Table 1) and their empirical relationships with seed yield (Table 2), the soybean literature remains fragmented regarding which traits should be prioritized for genetic improvement or agronomic optimization. These traits must be both biologically meaningful and heritable. To address this gap, we synthesized physiological mechanisms, phenotypic relationships and genetic evidence to propose a standardized core trait set that categorizes traits by biological scale, quantifies their documented effects on PE and yield components, and clearly defines their primary application domain (Table 3). This framework provides the biological foundation for the omics assisted breeding pipeline discussed below (Fig. 3).

**Table 3:** Core photosynthesis-related traits and proxies for soybean breeding: physiological roles, genetic architecture, and breeding relevance

Trait scale	Core trait(s)	Physiological bottleneck addressed	Quantified PE/yield effect	Genetic architecture & selectability	Primary breeding application	HTP platform/scalability
Leaf (instantaneous)	V_cmax25_, J_max25_	RuBP regeneration & carboxylation capacity	*J*_max25_negative genetic correlation with yield (r = − 0.62, SoyNAM); *PGK/PRK* overexpression projected +6.75% yield	Polygenic; Chr 10 (*FBPase*), *GmFtsH25*; moderate *H*^*2*^ at R2 (Table 1)	Genetic improvement	Low throughput; hyperspectral Chl proxies show moderate genetic correlation (r ~ 0.4–0.6)
	Stomatal conductance (*g*_s_)	CO_2_ diffusion limitation	Modern varieties show ~23% higher daily carbon assimilation via enhanced *g*_s_ under high moisture	High *H*^*2*^ at R2 (50–85%); QTLs *qP1*, *qP19*, *GmCDC48* (Table 1)	Genetic improvement	Thermal imaging; canopy temperature proxies
	Chlorophyll fluorescence (Fv/Fm, ΦPSII, NPQ)	PSII efficiency & photoprotection	Weak phenotypic yield correlation in some panels; transgenic NPQ relaxation claimed + ≤33% yield (contested)	Low-moderate *H*^*2*^ (37–47%); genes *GmFtsH25*, *GmRCA14, VDE* (Table 1)	Genetic improvement (stress resilience)	High (MultispeQ, LIFT, fluorescence imaging)
Canopy (integrated)	Chlorophyll content (SPAD, Chl a/b)	Light interception, N-status	Consistent moderate-strong yield correlation (r = 0.26–0.90); +0.26 phenotypic in SoyNAM	Variable *H*^*2*^; multiple QTLs; strong G×E at R6 (Table 1)	Integrated (Genetic + Management)	Very high (NDRE, hyperspectral indices)
	LAI, leaf angle, architecture	Light distribution, ε_i_	Historical gains in NE China linked to optimized architecture; excessive LAI causes self-shading	Moderate *H*^*2*^; architectural QTLs known (Table 1)	Integrated (Genetic + Management)	Very high (UAV structural indices, LiDAR)
	Canopy-apparent photosynthesis (CAP)	Cumulative carbon gain	Positive yield correlation (r = 0.28–0.66) at R4-R5	Limited field *H*^*2*^ data; linked to maturity loci (E2 region)	Management optimization (density, spacing)	Moderate (chamber-based); UAV proxies emerging
Non-foliar	Pod/seed photosynthesis	Source-sink carbon balance	Pod shading reduces seed weight 13–14%; offsets ~81% respiratory loss	Genetic architecture largely unexplored	Emerging target (both)	Very low; no established HTP proxy

This classification recognizes that trait deployment must match the source of variation. Leaf-level traits (fluorescence parameters, V_cmax25_, g_s_) typically show moderate to high heritability at early reproductive stages and have identifiable QTLs (Table 1). These are best suited for genetic improvement via genomic selection, allele introgression from diverse germplasm and transgenic validation. Canopy integrated traits such as CAP and optimal LAI are strongly modulated by planting density, row spacing and tillage. These are primary targets for management optimization to improve light distribution without genetic change. Bridge traits such as chlorophyll content and canopy architecture show both genetic variation and strong management responsiveness. These can be classified as integrated traits and are particularly valuable for multi-trait genomic prediction and G×E×M modeling, where genetic potential and management effects must be jointly optimized (Fig. 3). Non-foliar pod photosynthesis represents an emerging target for which both genetic architecture and scalable phenotyping remain to be explored.

Building on the core trait set, breeding programs must next ensure that selected traits are deployed at the appropriate biological scale and developmental stage. Traits contributing to RUE can be especially useful targets, provided they have an ample score of improvement (Faralli and Lawson 2020; Messina et al. 2022; Vialet-Chabrand 2024). Additionally, traits contributing to enhanced light interception, such as canopy architecture and optimization, have significant potential to be explored for photosynthetic improvements (He et al. 2024). These canopy-level traits primarily influence light interception (Ei) and canopy-level biomass accumulation (via RUE), whereas assimilate partitioning (HI) is often weakly related and is driven largely by sink strength, phenology, and stress during seed set and filling. Many canopy traits show moderate to high heritability in soybean (Table 2). However, seed yield is not always directly determined by photosynthetic traits. While photosynthetic traits primarily define intrinsic yield potential, their contribution to final seed yield depends on how effectively this potential is expressed under field conditions (White and Izquierdo 1989; Smith et al. 2018; Burgess and Degen 2023). Thus, selection for seed yield alone does not necessarily improve PE, whereas targeted selection for photosynthetic traits enables breeders to directly enhance physiological processes/traits that consistently underpin seed yield formation in specific environments.

Trait-yield relationships can differ substantially across soybean developmental stages (Table 2). For instance, PE measured at early reproductive stages may not reflect the same associations with yield as measurements taken later in the season, due to shifts in carbon partitioning and source-sink dynamics as pods and seeds become dominant sinks (Egli and Bruening 2001; Smith et al. 2018; Lopez et al. 2021). Capturing photosynthetic data at multiple stages, therefore, allows breeders to pinpoint when specific traits (e.g., canopy chlorophyll content, leaf-level CO_2_ assimilation, or canopy-level PE proxies) are most predictive of final seed yield. In practice, integrating PS-related measurements across developmental stages, rather than relying on a single time point, points to a more robust representation of season-long physiological performance and improves yield prediction. Several photosynthesis-related traits, including leaf morphology, chlorophyll status, and stomatal traits, often show higher heritability than seed yield and are relatively less influenced by G**×**E interactions (Richards 2000; Acevedo-Siaca et al. 2020; Silva-Pérez et al. 2020), making them valuable for indirect selection and early prediction modeling. Moreover, identifying which PE-related traits are limiting in high or low-yielding germplasm can guide both genetic improvement and agronomic optimization.

Once target traits are prioritized, the next step is to identify novel genetic resources with useful variation. The United States Department of Agriculture (USDA) Soybean Germplasm Collection, part of the National Plant Germplasm System (NPGS), represents one of the largest publicly available soybean diversity resources. It comprises ~ 20,000 accessions of cultivated and wild soybean originating from more than 80 countries worldwide. Importantly, a large proportion of this collection has been genotyped using high-density SNP platforms (e.g., SoySNP50K), enabling breeders to select germplasm based on genome-wide diversity rather than relying solely on maturity group or geographical origin. Screening these panels using HTP platforms- such as UAV-based multispectral imaging, chlorophyll fluorescence sensors, or canopy-level gas exchange systems enables evaluation of photosynthetic traits across multiple growth stages and environments. However, variation in RUE or its proxies identified in diverse or exotic germplasm should not be interpreted as direct seed yield advantage in elite backgrounds, as favorable alleles are often embedded in poorly adapted genetic backgrounds. Instead, the primary value of large-scale screening lies in identifying physiologically superior alleles and trait expressions, that are specifically useful in a pre-breeding context. They can be introgressed into elite germplasm to improve PE without compromising adaptation. Accurate phenotyping of prioritized photosynthetic traits under dynamic field conditions remains essential for capturing real-world variability and ensuring their relevance to breeding objectives (Song and Zhu 2024). Moreover, plot-level canopy photosynthesis provides amore integrative measure of a genotypic performance compared to individual leaf-level measurements (Theeuwenet al. 2022).

Identifying phenotypic and genotypic correlations between photosynthetic traits and seed yield under field conditions is a key step toward translating physiological knowledge into breeding outcomes (Lopez et al. 2019; Montes et al. 2022; Hu et al. 2024; Keller et al. 2024). To translate PE into breeding gains, programs should emphasize traits that combine physiological relevance with scalability, including RUE, canopy-level photosynthetic proxies (e.g., SIF, NDRE, PRI), biomass accumulation, and dynamic photoprotective responses underfluctuating light. These traits are not selected from single measurements, but are phenotyped using UAVs-based multispectral, hyperspectral, thermal, and fluorescence platforms, calibrated against detailed physiological measurements in smaller training populations. Because seed yield is strongly influenced by environment and G×E, the incorporation of photosynthesis-related traits into multi-trait genomic prediction models enables breeders to capture physiological variation that yield alone does not reveal, improving prediction accuracy and selection efficiency (Lopez et al. 2019; Montes et al. 2022).

USDA soybean accessions and diverse germplasm panels play a central role in this framework by providing the genetic diversity needed to identify favorable or previously lost alleles for PE and RUE. These panels are best used to train genomic-phenomic prediction models, characterize trait architecture, and identify donor haplotypes rather than as direct yield-improving varieties. Favorable alleles identified in unadapted germplasm can then be introgressed into elite backgrounds through pre-breeding pipelines, including genomics-informed donor selection, strategic cross-design (elite×donor), marker or haplotype-assisted introgression, and genomic selection to simultaneously recover elite background while retaining target photosynthetic traits during early-generation advancement. Within breeding programs, phenomics and genomics should be integrated across defined stages: model development in diverse panels, early-generation selection for PE traits using genomic prediction and HTP-derived proxies, and combined selection for yield potential and stability in advanced multi-environment trials. Together, this framework allows systematic incorporation of photosynthesis-related traits into soybean breeding programs aimed at improving intrinsic yield potential.

Several studies illustrate the potential of these approaches. Lopez et al.(2019) reported a strong genetic correlation (0.80) between seed yield and photosynthesis (gas exchange based) and showed high predictive ability for photosynthesis using genomic prediction (accuracy > 0.65), supporting genomic prediction as a route to overcome the phenotyping bottleneck. Recent work by Keller et al. (2022) demonstrated moderate accuracy (~ 0.50) in predicting soybean biomass using a dynamic photosynthetic response index (Response_G:PPFR_), that captures the interaction between chlorophyll fluorescence (Fq’/Fm’) and fluctuating light. Extending this approach in common bean (*Phaseolus vulgaris* L.), Keller et al. (2024) applied both genomic and phenomic prediction (PP) to model seed yield using photosynthetic traits. Despite a relatively low but significant correlation (r = 0.26) between response Response_G:PPFR_ and seed yield across multiple field trials, PP outperformed genomic prediction.

Traditional GP estimates breeding values from marker-phenotype relationships in a training population. However, this approach imposes structural constraints for photosynthetic traits. Prediction accuracy depends strongly on genomic relatedness between training and target populations. When target population is genetically distant from the training set, accuracy declines sharply and selection is biased toward elite germplasm (Rincent et al. 2017; Isidro y Sánchez et al. 2021). Furthermore, standard genomic best linear unbiased prediction captures main genetic effects but remains largely indifferent to environment-specific expression unless explicitly modeled. Photosynthetic traits are highly plastic. Their genetic expression is modulated by light intensity, temperature, and water availability. Without environmental covariates, GP cannot capture this plasticity. A third limitation concerns early breeding generations. GP requires phenotypic records on the training population. In early generations, yield data are scarce and photosynthetic traits have not been measured at scale. This restricts deployment for PE-based selection when decisions are most impactful.

Single-time-point phenotyping is equally limiting for photosynthetic traits. These traits vary diurnally and developmentally. Stomatal conductance and non-photochemical quenching change within hours (sometimes even minutes). Carboxylation capacity and chlorophyll content shift across vegetative and reproductive stages. A measurement taken during transient midday stress or late-season senescence may reflect environmental noise rather than intrinsic genetic potential. Hu et al. (2024) reported that heritability of chlorophyll fluorescence parameters dropped from 90.8% at the R2 stage to 16.8% by R6. This means a single late-season measurement would miss most of the genetic signal. Conventional phenotypic selection, which typically relies on one or two measurements per season, therefore risks selecting on ephemeral environmental responses rather than stable genetic differences.

Phenomic prediction addresses several of these limitations. It uses high-throughput phenotyping proxies such as spectral indices, fluorescence parameters, or canopy temperature to predict yield or biomass without waiting for harvest. This shortens breeding cycles and allows screening of thousands of early-generation plots in a single season. Krause et al. (2020) demonstrated that aerial high-throughput phenotyping enables indirect selection for grain yield at seed-limited stages in wheat breeding programs. Unlike conventional phenotypic selection, which averages performance across environments after harvest, PP models are trained on environment-specific HTP data. They therefore implicitly capture G×E interactions that conventional selection misses.

Phenomic prediction also offers distinct advantages over stand-alone genomic prediction. It leverages observed, environment-responsive phenotypes. Canopy reflectance, thermal signatures and fluorescence integrate genetic potential with its actual field expression. GP, on the other hand, estimates genetic potential from DNA alone. It cannot know whether that potential was realized under specific growing conditions unless environmental covariates are explicitly included. Keller et al. (2024) showed that PP outperformed GP for seed yield in common bean despite a low direct correlation between the photosynthetic index and yield. van der Laan et al. (2025) similarly reported that combining phenomic and genomic prediction improved accuracy for soybean seed yield, protein, and oil. For early-generation screening, phenotyping with UAVs or portable sensors is often cheaper and faster than genotyping thousands of individual plants. PP is therefore particularly valuable for photosynthetic traits that are environmentally sensitive and not fully captured by genomic information alone.

Soybean traits suitable for phenomic modeling fall into three scalable categories. Canopy spectral indices derived from UAV multispectral or hyperspectral imaging provide proxies for chlorophyll content, leaf area index, and canopy vigor. Montes et al. (2022) showed that hyperspectral reflectance is genetically correlated with V_cmax_ and J_max_ in the SoyNAM population. Red-edge and near-infrared indices predict plant height, stomatal conductance, and yield with high accuracy when coupled with machine learning. Chlorophyll fluorescence parameters including Fv/Fm and ΦPSII can be captured via ground-based platforms such as MultispeQ or light-induced fluorescence transient meters. These directly proxy photosystem II efficiency and photoprotective status. Canopy temperature from UAV thermal imaging provides an indirect estimate of stomatal conductance and transpiration, both tightly linked to PE and WUE. Dynamic indices that capture responses to fluctuating light offer predictive power beyond static single-state measurements. Finally, canopy structural traits including plant height, LAI, and canopy coverage integrate light interception efficiency and are readily extracted from spectral or LiDAR data.

Early-generation germplasm screening is the most immediate application of phenomic prediction in soybean breeding. The workflow proceeds in three phases. First, a diverse training population is phenotyped with HTP platforms and yield data across multiple environments. Second, a prediction model is calibrated relating spectral, thermal, or fluorescence proxies to yield or biomass. Third, in early generations when seed is too limited for replicated yield trials, thousands of F_2_ or F_3_ lines are screened with UAVs at R2 to R5–R6. The model predicts yield potential for each line. Only the top ten to twenty percent are advanced to multi-environment yield trials. Krause et al. (2020) demonstrated that aerial HTP enables indirect selection for grain yield at exactly these seed-limited stages in wheat. This approach conserves resources and compresses selection cycles.

Multi-environment yield forecasting extends PP from selection to within-season decision support. Time-series phenotyping at 2–3 key reproductive stages captures developmental dynamics that single measurements miss. Spectral/thermal data collected across multiple locations are integrated with seasonal environmental covariates including temperature, humidity, radiation, and soil moisture. Reaction-norm or mixed models then predict yield at each location before harvest. Jarquín et al. (2014) showed that incorporating environmental covariates into prediction frameworks enhances accuracy across diverse environments. For soybean, this allows breeders to distinguish genotypes that maintain high predicted yield across environments from those that excel only under specific conditions. Phenomic prediction therefore supports both selection and stability assessment.

Neither genomic nor phenomic prediction alone is sufficient for photosynthetic trait-based breeding. GP remains valuable for identifying favorable alleles in exotic germplasm and guiding parental selection. PP excels at estimating environment-responsive field performance in early generations. A complementary strategy that uses GP to uncover genetic potential and PP to estimate realized performance offers the most scalable pathway. van der Laan et al. (2025) demonstrated this complementarity for soybean seed yield, protein, and oil. By embedding phenomic traits as secondary predictors within multi-trait genomic selection frameworks, breeders can improve prediction accuracy while reducing reliance on costly multi-environment yield trials.

Promising genotypes can then serve as (1) donor parents for population development that combines high seed yield with enhanced PE, and (2) training populations for predictive modeling. GWAS supports this framework by identifying candidate loci/genes and guiding allele mining from diverse germplasm (Hu et al. 2024; Ali et al. 2025; Sansa et al. 2024). Genomic selection (GS) complements this by leveraging genome-wide marker information to capture the small effects of many loci simultaneously (Croce et al. 2024; Faralli and Lawson 2020), which is essential for complex photosynthetic traits. Together, GWAS and GS integrate biological understanding with practical prediction for improving soybean productivity.

Genetic improvement alone cannot fully realize PE potential. Management practices are active determinants of photosynthetic efficiency. They interact with genotype and environment to modulate PE trait expression. These practices are not passive backdrops for genetic expression. Canopy architecture illustrates this interaction clearly. Narrow, erect leaves reduce upper canopy saturation and improve penetration to lower leaves. However, such genotypes achieve higher RUE only at appropriate planting densities and row spacings. Excessive shading increases LAI beyond optimal leaves and negates the architectural advantage through self-shading (Tamang et al. 2026; Sreekanta et al. 2024; Srinivasan et al. 2017). Stomatal conductance provides another example. Modern varieties with higher stomatal conductance exhibit greater daily carbon assimilation under ample soil moisture (Koester et al. 2016). Under water limiting conditions, this advantage diminished unless supported by residue retention, cover cropping or irrigation (Whippo et al. 2024).

Several management factors systematically alter PE. Planting density and row spacing modulate canopy light interception and self-shading (Sreekanta et al. 2024). Tillage and residue management modify soil temperature, moisture, and early canopy development (Buczek et al. 2022; Wang et al. 2022b). Cover crops improve WUE and PE under drought (Whippo et al. 2024). Nutrient management, including nitrogen, phosphorus, biochar and organic amendments, affects chlorophyll content, Rubisco activity and LAI (Qiang et al. 2023; Zhao et al. 2022; Wu et al. 2024; Dossin et al. 2024). Irrigation timing is especially critical during reproductive stages when seed set and filling drive carbon demand (Wang et al. 2022b). Weed control prevents light competition and shade avoidance responses that suppress canopy photosynthesis (Swanton et al. 2015; Millar et al. 2011; Horvath et al. 2015).

These factors are not background conditions, rather they determine whether genetic potential for PE is realized. A genotype with high intrinsic RUE may still yield poorly if planted at excessive density. Weed competition can similarly compromise N_2_-fixation and thereby PE. Therefore, management parameters must be co-optimized with genotype under target environments. Factorial G×E×M trials and environmental covariates in prediction models are essential (Cooper et al. 2021; Hajjarpoor et al. 2022). This integration prevents selection of genotypes whose PE advantages depend on poorly managed field conditions.

A key next step is integrating HTP, genomic, and environmental data to connect photosynthetic traits with predictive breeding frameworks. Multi-trait genomic prediction models that incorporate photosynthesis-related traits as secondary predictors have shown improved accuracy over single-trait models, particularly when secondary traits exhibit higher heritability and genetic correlations with yield (Crossa et al. 2017; Montesinos-López et al. 2022). In parallel, incorporating environmental covariates using reaction-norm or G×E models has been demonstrated to enhance prediction across diverse environments compared to baseline genomic best linear unbiased prediction (GBLUP) approaches (Jarquín et al. 2014; Cooper et al. 2021). In soybean, strong genetic correlations between gas exchange traits and yield (r ≈ 0.80) and moderate prediction accuracy for dynamic photosynthetic indices (Keller et al. 2022; 2024; Lopez et al. 2019) support their integration into multi-trait frameworks. Similarly, high-throughput phenomic traits have improved prediction accuracy in several crops by capturing environment-responsive physiological variation not explained by markers alone (Araus and Cairns, 2014; Crain et al. 2018).

Together, these findings indicate that while baseline GBLUP remains robust, integrating physiologically relevant traits and environment covariates (e.g., seasonal radiation, soil moisture, or temperature profiles) can provide incremental but consistent gains in predictive ability. This is particularly relevant for photosynthesis-driven breeding, where traits are dynamic, environment-sensitive, and not fully captured by genomic information alone. In practice, these models can prioritize promising genotypes before large-scale yield trials, especially in early breeding stages or off-season nurseries where direct yield measurement is limited, thereby improving selection efficiency and resource allocation. This supports selection of genotypes that combine favorable PE with stable yield performance across target production regions.

Integrating these diverse datasets requires a structured, stepwise approach that accounts for differences in scale, dimensionality, and biological rationale. Refining models further demands explicit representation of G**×**E**×**M interactions to account for context-dependent trait expression. This can be achieved by (1) embedding high-dimensional environmental data (e.g., soil moisture, temperature fluxes, radiation, precipitation) as covariates within multi-trait genomic prediction frameworks, scaling inputs via feature selection or latent variable decomposition such as environmental PCA; (2) applying hybrid model structures (e.g., coupling mechanistic crop models with machine learning approaches to dynamically link photosynthetic traits to seed yield under management scenarios); or (3) employing causal inference approaches (e.g., structural equation modeling) to disentangle direct and indirect effects of photosynthetic traits on seed yield across environments.

Crucially, all prediction frameworks must be validated across target environments to ensure model robustness and transferability. High-dimensional phenomic data (e.g., spectral indices, fluorescence outputs) should be standardized and condensed into biologically interpretable latent variables, enabling integration with genomic markers and environmental covariates in multi-trait models. This validation-focused workflow allows breeders to prioritize genotypes that consistently combine favorable PE with yield stability, reducing reliance on costly multi-environmental trials.

Ultimately, photosynthetic trait-based breeding should not be viewed as a replacement for direct seed yield selection, but as a complementary strategy. Physiological traits such RUE and its scalable proxies provide early, heritable, and biologically interpretable indicators that can improve prediction accuracy, guide parental selection and optimize resource allocation (Fig. 3). By embedding these traits within genomic prediction pipelines and validating their performance across target environments, soybean breeders can systematically integrate PE into yield improvement strategies while keeping seed yield as the ultimate selection criterion.

## Future directions

Future progress in photosynthesis-informed soybean breeding will require moving beyond mainly additive, single-trait frameworks toward approaches that better represent biological complexity. Although GWAS have identified numerous loci associated with PE (Ali et al. 2025; Hu et al. 2024; Montes et al. 2022), most signals reflect marginal marker effects and remain functionally unvalidated. A priority is rigorous validation of candidate loci using functional genomics (e.g., CRISPR/Cas and transgenics), followed by multi-environment field testing to confirm agronomic relevance and stability across genetic backgrounds.

A major frontier is resolving true epistasis and nonlinearity in PE architecture. Photosynthesis likely reflects coordinated gene networks spanning photochemistry, carbon fixation, source-sink regulation, and stress signaling (Mackay 2014; Wang et al. 2018). In such scenario, gene–gene interactions may be important but are rarely modeled explicitly in standard GWAS and GP pipelines (Crossa et al. 2017). Emerging computational approaches particularly deep learning, graph neural networks, and causal inference frameworks offer opportunities to capture nonlinear interactions and identify subnetworks that jointly control canopy-scale carbon gain (Montesinos-López et al. 2021; Van Dijk et al. 2021a, b; Washburn et al. 2021). The near-term opportunity is not “black-box prediction,” but using these methods to propose testable interaction hypotheses that can be validated experimentally. Beyond task specific deep learning, large language models (LLM) and foundation models represent an emerging computational frontier. Pre-trained DNA language models and vision transformers trained on global remote sensing archives are beginning to enable cross-species genomic prediction and low data yield forecasting (Mendoza-Revilla et al. 2024). Their capacity to integrate heterogeneous genotypic, phenotypic, and environmental datasets may eventually offer a route to capture complex nonlinear G×E×M interactions that elude simpler statistical frameworks, although direct application to complex photosynthetic traits remains in its infancy.

Another underdeveloped area is directly linking PE with biotic and abiotic stress resilience, rather than treating them as separate breeding targets. Drought, heat, flooding, and soil-borne pathogens constrain PE through different mechanisms, influencing stomatal conductance, mesophyll diffusion, electron transport, and carbohydrate partitioning (Flexas et al. 2014; Renziehausen et al. 2024). Future work should quantify how stress-adaptive traits reshape PE dynamics (maintenance, plasticity, and recovery), especially during reproductive stages when seed set and filling dominate carbon demand. This includes testing whether ‘stress tolerant’ soybean genotypes maintain PE via conservative strategies that protect photochemistry but cap peak assimilation, and identifying genetic architectures that allow rapid recovery after stress events.

Current GP pipelines also impose structural limits on PE improvement because prediction accuracy depends strongly on genomic relatedness between training and target populations. This can bias selection toward elite germplasm and reduce the probability of discovering useful alleles in genetically distant accessions (Rincentet al. 2017; Isidro y Sánchez et al. 2021). Future work should emphasize training set design as a research problem- physiologically-informed training populations that maximize PE-related diversity, multi-parent designs, and strategic inclusion of exotic donors can improve transferability and reduced “genomic-distance exclusion” (Gorjanc et al. 2016). These designs are especially relevant when the goal is to recover lost alleles for RUE and canopy photosynthesis from landraces or wild relatives.

HTP should also shift from static trait snapshots to dynamic characterization of PE, including diurnal trajectories, developmental changes, and post-stress recovery kinetics. UAVs-based hyperspectral, thermal, and fluorescence imaging already provides the plot-level resolution needed for time-series phenotyping in breeding trials (Borra-Serranoet al. 2020; Khuimphukhieo and da Silva 2025). Satellite remote sensing is not yet sufficient for plot-scale breeding due to spatial resolution limits. However, improvements in resolution and revisit frequency could eventually enable hybrid satellite-UAVs workflows that lower cost and expand spatial coverage. Linking time-series phenotypes to crop growth models that explicitly represent photosynthetic parameters (e.g., V_cmax_, J_max_, mesophyll conductance) offers a path to hybrid prediction where physiology contains statistical learning, enhancing predictive power (Wu et al. 2019; Hammer et al. 2019).

Standardization is a practical bottleneck. Lack of consistency in growth stages, measurement conditions, and trait definitions limits cross-study comparability and slows breeding translation. Community-agreed protocols for field phenotyping for PE-related traits, paired with open benchmark datasets for model comparison, would accelerate progress and reduce reproducibility concerns, especially for AI-based models. However, the application of AI in breeding also raises practical and ethical considerations. Complex models risk becoming opaque, limiting biological interpretability and reproducibility, while data imbalance and population structure may amplify existing biases toward elite germplasm (Van Dijk et al. 2021a, b). Addressing these challenges will require transparent model architectures, open benchmarking datasets, and explicit evaluation of fairness and transferability across genetic backgrounds and environments.

Finally, nitrogen-photosynthesis interactions deserve targeted experimental work in soybean because biological N-fixation complicates inference. N-deficiency reduces chlorophyll content, Rubsico activity, and CO_2_ assimilation (Qiang et al. 2023), but future research should leverage non-nodulating isolines and controlled N regimes to quantify causal effects of N-supply on PE and yield formation. This would clarify whether PE improvements are best pursued through canopy/leaf traits alone or through integrated strategies that also optimize N-acquisition, allocation, and seasonal canopy duration.

At the systems level, integrating transcriptomics, proteomics, metabolomics, and epigenomics with metabolic flux modeling and AI-driven inference offers a route to identify regulatory control points inaccessible through single-layer analyses (Lopez et al. 2019; Keller et al. 2022; Montes et al. 2022). Rather than optimizing individual traits, future work should aim to characterize coordinated genetic networks controlling carbon assimilation, photoprotection, and source-sink dynamics, and evaluate how these networks respond to environmental stress. This shift toward network-level understanding may allow breeding strategies that target robustness, plasticity, and stability of PE across environments.

Importantly, these approaches must be coupled with standardized field phenotyping, well-designed training populations, and multi-environment validation to ensure that system-level insights translate into measurable gains in seed yield. Without this integration, advances in multi-omics and AI will remain descriptive rather than actionable for soybean breeding.

## Conclusion

Soybean yield improvement through PE represents both a long-standing challenge and a promising opportunity. Decades of research have established that photosynthesis is not only a driver of biomass production but also an integrator of genetic, physiological, and environmental processes that determine seed yield. Empirical studies have shown significant associations between photosynthetic parameters such as chlorophyll content, Fv/Fm, ΦPSII, net CO_2_ assimilation rate, and seed yield across diverse environments (Liu et al. 2012; Hu et al. 2024; Montes et al. 2022). However, direct selection for photosynthetic traits has historically been constrained by their often low heritability and the difficulty of measuring them at a cultivar development pipeline scale.

In soybean and other C3 crops, RUE and related processes are not yet theoretically maximized and optimized, while substantial natural genetic variation exists within global germplasm collections. At the same time, genetic and physiological studies are increasingly revealing the complex architecture of these traits, providing new entry points for breeding. Although transgenic approaches have demonstrated that targeted manipulation of photosynthetic pathways can enhance PE and biomass accumulation, consistent translation to stable seed yield benefits under field conditions remains limited, reinforcing the need for integration with standard breeding and agronomic evaluation. Recent advances in HTP, genomics, and multi-trait modeling frameworks are reshaping this landscape to incorporate photosynthesis into breeding pipelines. The integration of GS and GWAS with dynamic, physiologically meaningful photosynthetic traits enables improved prediction of yield potential and stability across environments, including explicit consideration of G×E×M interactions (Croce et al. 2024; Ali et al. 2025; Sansa et al. 2024). Scalable phenotyping platforms, including UAVs-based imaging, hyperspectral reflectance, and chlorophyll fluorescence, now provide practical proxies for PE that can be deployed at a population scale.

Ultimately, the success of photosynthesis-informed breeding will depend on collaborative, interdisciplinary efforts that combine physiological insights, genetic diversity, and predictive analytics within applied breeding programs. By embedding photosynthetic traits as complementary selection criteria alongside yield, breeders can more precisely target intrinsic yield potential while maintaining agronomic performance and stability. With continued refinement of phenotyping, modeling, and field validation strategies, the long-standing goal of improving soybean yield through photosynthesis optimization can progress from conceptual promise to practical realization.

## Data Availability

No datasets were generated or analyzed during the current study.
